# Effect of 2′-5′/3′-5′ phosphodiester linkage heterogeneity on RNA interference

**DOI:** 10.1093/nar/gkaa222

**Published:** 2020-04-13

**Authors:** Maryam Habibian, S Harikrishna, Johans Fakhoury, Maria Barton, Eman A Ageely, Regina Cencic, Hassan H Fakih, Adam Katolik, Mayumi Takahashi, John Rossi, Jerry Pelletier, Keith T Gagnon, P I Pradeepkumar, Masad J Damha

**Affiliations:** 1 Department of Chemistry, McGill University, 801 Sherbrooke St. West, Montreal, QC H3A 0B8, Canada; 2 Department of Chemistry, Indian Institute of Technology Bombay, Mumbai 400076, India; 3 Department of Biochemistry and Molecular Biology, Southern Illinois University School of Medicine, Carbondale, IL, USA; 4 Department of Chemistry and Biochemistry, Southern Illinois University, Carbondale, IL, USA; 5 Department of Biochemistry and Goodman Cancer Center, McGill University, Montreal, QC H3G 1Y6, Canada; 6 Department of Molecular and Cellular Biology, Beckman Research Institute of City of Hope, Duarte, CA, USA

## Abstract

We report on the synthesis of siRNAs containing both 2′-5′- and 3′-5′-internucleotide linkages and their effects on siRNA structure, function, and interaction with RNAi proteins. Screening of these siRNAs against their corresponding mRNA targets showed that 2′-5′ linkages were well tolerated in the sense strand, but only at a few positions in the antisense strand. Extensive modification of the antisense strand minimally affected 5′-phosphorylation of the siRNA by kinases, however, it negatively affected siRNA loading into human AGO2. Modelling and molecular dynamics simulations were fully consistent with these findings. Furthermore, our studies indicated that the presence of a single 5′p-rN_1_-(2′-5′)-N_2_ unit in the antisense strand does not alter the ‘clover leaf’ bend and sugar puckers that are critical for anchoring the 5′-phosphate to Ago 2 MID domain. Importantly, 2′-5′-linkages had the added benefit of abrogating immune-stimulatory activity of siRNAs. Together, these results demonstrate that 2′-5′/3′-5′-modified siRNAs, when properly designed, can offer an efficient new class of siRNAs with diminished immune-stimulatory responses.

## INTRODUCTION

**Figure 1.**
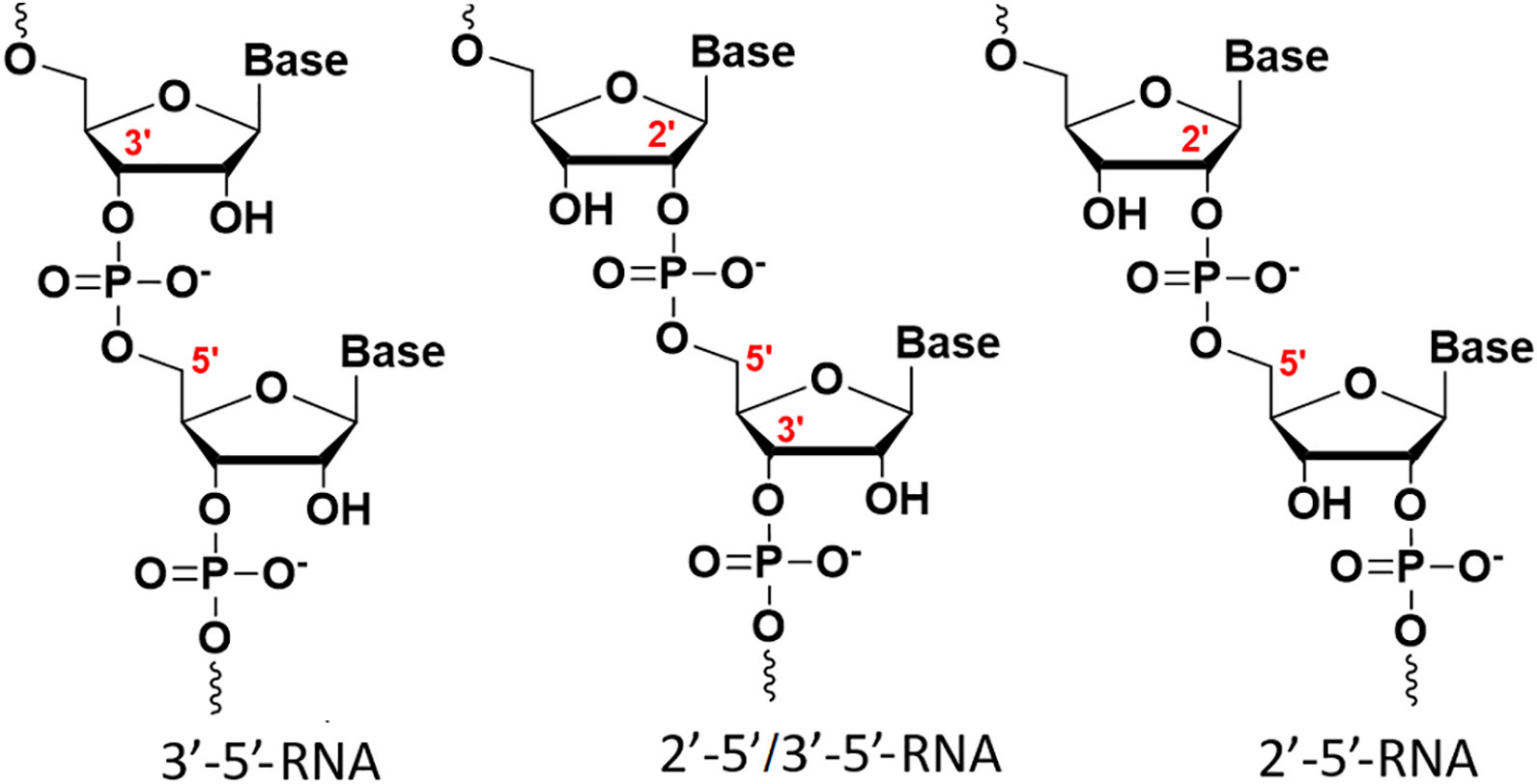
Structures of regioisomeric RNAs.

RNA is proposed to have played a key role in the early evolution of primitive life before DNA and proteins evolved (1,2). In such a hypothetical ‘RNA world’, RNA served as both the genetic material and as the principal catalyst of essential biochemical reactions (3). In accordance with this theory, nonenzymatic RNA replication would conceivably lead to a mixture of 2′-5′ and 3′-5′ linkages, and this raised questions about the role and relevance of backbone heterogeneity in early-life RNA replication (Figure 1) (4). Recent research by Szostak *et al.* demonstrated that formation of 2′-5′ linkage may have been vital for the development of a prebiotic world (5). As the presence of 2′-5′-linkages lowers the melting temperature of RNA duplexes, allowing copied strands to dissociate (6,7), backbone heterogeneity may have been an essential feature that allowed RNA to emerge as the first biopolymer (5). Recent high resolution crystallographic data on mixed-backbone RNA duplexes show that RNA duplexes containing a few 2′-5′-linkages share the same global A-like structure as the native duplex, suggesting that RNA duplexes can accommodate perturbations caused by these linkages (8,9). Remarkably, RNA helical structures are well retained with 40% backbone heterogeneity (9). The potential role of 2′,5′-linkages in early RNA metabolism serves as an inspiration for why these linkages should be tested and why they might function in RNA interference (RNAi), a biological process in which RNA molecules inhibit gene expression by targeting mRNA molecules.

An earlier study showed that siRNAs with an entirely 2′-5′-linked sense strand possessed better, albeit moderate, activity compared to an siRNA with an entirely 2′-5′-linked antisense strand (10). Thus, it was concluded that the 2′-5′ linkage could only be useful as a ‘sense-only’ modification in siRNAs (10), while partially modified antisense or sense strands were not studied. In this study, we have synthesized and screened several siRNAs in human cells targeting luciferase, P53 and debranching enzyme 1 (Dbr1) mRNA with varying 2′-5′/3′-5′ content and positional variations throughout both sense and antisense strands. Using *in vitro* assays and molecular modeling, we also investigated the interactions of these modified siRNAs with enzymes involved in the RNAi pathway, specifically kinases and human AGO2 (hAGO2). The immune-stimulatory effects of a small set of duplexes were also assessed by measuring IFN-α and IL6 levels in PBMCs. The data shows that 2′-5′ linkages are tolerated at certain positions of both the antisense (guide) and sense (passenger) strand and can provide siRNAs with significantly diminished immune-stimulatory responses.

## MATERIALS AND METHODS

### Synthesis and purification of canonical, modified and mixmer oligonucleotides

Standard phosphoramidite solid-phase synthesis conditions were used for the synthesis of all modified and unmodified oligonucleotides (11). Syntheses were performed on an Applied Biosystems 3400 DNA Synthesizer at a 1-μmol scale using Unylinker CPG support (ChemGenes). Oligonucleotides containing either 3′-5′ or 2′-5′ RNAs were both synthesized with standard 2′-TBDMS and 3′-TBDMS phosphoramidites respectively. All phosphoramidites were prepared as 0.15 M solutions in acetonitrile (ACN) to maximize coupling efficiency. Mixmer RNAs were generally prepared from 1:1 amidite mixtures of the 2′ and 3′ phosphoramidites, unless stated otherwise. 5-Ethylthiotetrazole (0.25 M in ACN) was used to activate phosphoramidites for coupling. Control experiments utilizing varying molar ratio mixtures of 5-Me-rU 3′-amidite and rU 2′-amidite (to help differentiate mixmers by MS) showed that the 3′ and 2′ amidites coupled with *relative* efficiencies of 1 to 0.77, respectively (Supplementary Figure S1). Hence, our 1:1 mixmer RNAs were slightly enriched with 3′-5′ linkages. Detritylations were performed using 3% trichloroacetic acid in CH_2_Cl_2_ for 110 s. Failure sequences were capped using acetic anhydride in THF and 16% N-methylimidazole in THF. A 0.1 M solution of I_2_ in 1:2:10 pyridine:water:THF was used for oxidation. Coupling times were 600 seconds except for the guanosine phosphoramidites which were coupled for 900 s. Chemical 5′-phosphorylation of selected modified guide strands was done using bis-cyanoethyl-*N*,*N*-diisopropyl-2-cyanoethyl phosphoramidite at 0.15 M (600 s coupling time). Deprotection and cleavage from the solid support for all samples was accomplished with 3:1 NH_4_OH:EtOH for 48 h at room temperature (for 2′-5′ modified samples), and with 40% methylamine for 10 min at 65°C (for unmodified RNA samples) (12). In principle, 2′-5′ modified samples could be deprotected with 40% methylamine (10 min, 65°C), however, we chose to follow our previously published protocols (11). Desilylation of all samples was achieved with triethylamine trihydrofluoride/*N*-methyl pyrrolidone/triethylamine (1.5:0.75:1 by volume) for 2.5 h at 65°C (12,13).

Purification of crude oligonucleotides was done by HPLC using a Waters Protein-Pak DEAE 5PW anion exchange column (21.5 × 150 mm) and a buffer system consisting of water (solution A) and 1 M lithium perchlorate solution in water (solution B) at 0−40% gradient of B over 40 min at 60°C. The desired peaks eluted at 20−30 min. The collected samples were lyophilized to dryness and were desalted with Sephadex columns from GE Healthcare. Purified oligonucleotides were then lyophilized to dryness and were characterized by ESI-mass spectrometry in negative mode (Supplementary Table S1). Sequences were verified by high resolution ESI-LCMS in negative mode. Since 2′-5′ linked RNA is a regioisomer of RNA, all 2′-5′ modified samples and mixmers had the same mass as their corresponding unmodified control. siRNAs duplexes were prepared by annealing equimolar quantities of complementary oligonucleotides in siRNA buffer (100 mM KOAc, 30 mM HEPES–KOH, 2 mM Mg(OAc)_2_, pH 7.4) by slowly cooling from 96°C to room temperature, and then keeping them at 4°C overnight.

### Thermal denaturation experiments and circular dichroism studies

UV thermal denaturation data of all siRNAs were obtained on a UV-VIS spectrophotometer equipped with a Peltier temperature controller. Duplex concentration for all siRNAs, was 1.5 μM (3 μM total concentration of single strands) in 140 mM KCl, 1 mM MgCl_2_ and 5 mM Na_2_HPO_4_ (pH 7.2–7.4). The temperature was increased at a rate of 0.4°C/min from 5°C to 95°C for all duplexes. Absorbance values at 260 nm were monitored every minute upon cooling and heating steps. Samples were kept under nitrogen flow when below 15°C. *T*_m_ values were calculated using the heating ramp and baseline method.

CD spectra were obtained at a duplex concentration of 5 μM (10 μM total concentration of strands) at a range of temperatures. Samples were slowly annealed (cooling from 95 to 4°C over several hours) prior to recording their CD spectra in 140 mM KCl, 1 mM MgCl_2_ and 5 mM Na_2_HPO_4_ (pH 7.2). Spectra were recorded at 25°C under constant flow of nitrogen gas and was baseline-corrected with respect to a blank containing the buffer. Smoothing and adjustment for duplex concentration were performed using the Spectra-Manager program (Jasco) (Supplementary Figure S2).

### Luciferase siRNA knockdown assay

Luciferase knockdown assays were performed as described in literature (14), with a few modifications. HeLa cells were counted and seeded at a density of 10 000 cells/well in a 96-well plate and were incubated for 24 h at 37°C with 5% CO_2_ prior to the experiment. Subsequently, cells were washed once with serum-free DMEM media and then 80 μl of serum-free DMEM media was added. siRNA and control duplexes were diluted up to 20 μl with serum-free media and transfection reagent (Oligofectamine, Invitrogen) and added to the appropriate well (for a total of 100 μl) at increasing concentrations (0.16, 0.8, 4, 20 and 100 nM). Cells were incubated overnight (for a total of 24 h post-transfection). Then 50 μl of ONE-Glo luciferase reagent (Promega, USA) was added to each well and luminescence was measured and normalized to protein levels using a Biotek Synergy HT plate reader.

### P53-renilla reporter plasmid cloning

Cloning of a P53 reporter was performed following the instructions reported in literature (15). A G-block containing the P53 target site flanked by restrictions sides for XhoI and NotI at the ends of the sequence (IDT Technologies) was used to clone the sequence into the 3′ UTR of the renilla gene of plasmid PsiCheck2 (Promega), a plasmid also containing hluc+ (a synthetic firefly luciferase gene) and which conveniently served as internal transfection control. Following the cloning, the insert was sequence verified and found to be: 5′-atccgtttcaagccgCTCGAGagcgtggtggtaccttatgagccacccgaggccg gctctgagtataccaccatccactacaagtacatgtgtaatagctcctgcatggggggcatgaaccgccgacctatccttaccatcGCGGCCGCgttcataggcttatg-3′.

### P53-renilla reporter assay

Cells (293T) were seeded with 5000 cells/well in a 96-well plate. After 12 h, they were transfected with the siRNAs using Oligofectamine (Invitrogen) and Ca_3_(PO_4_)_2_. Twenty-four hours after siRNA transfection, cells were transfected with 20 ng/well of the bi-cistronic hRLuc/hluc+ plasmid, using Ca_3_(PO_4_)_2_. Forty-eight hours after the plasmid transfection, cells were harvested in 100 μl PLB (Passive Lysis Buffer, Promega) and 5 μl was used to read FF/Ren using a Fluorostar Optima plate reader. Experiments were repeated in three biological replicates.

### Dbr1 siRNA knockdown assay

HeLa cells were seeded at 150 cells/well in six-well dishes and transfected 24 h later with siRNAs at the indicated final concentrations using RNAiMAX (Invitrogen) following the manufacturer's recommended protocol. At 48 h post-transfection, total RNA was extracted from cells by adding 1 ml of TRIzol reagent (Invitrogen) to each well, pipetting up and down, then transferring to microfuge tubes. Chloroform was added (0.2 ml) and the mixture shaken, centrifuged and the top aqueous layer collected and precipitated with 1 volume of isopropanol. Extracted RNA (2 μg) was converted to cDNA by first eliminating contaminating DNA with 10 units DNase I (Worthington), heat inactivating at 70°C for 10 min, then generating cDNA using the Hi-Capacity cDNA Synthesis Kit (Applied Biosystems) as per the manufacturer's recommended protocol. Quantitative SYBR PCR was performed with 20 ng of cDNA using validated primer sets against the indicated genes. Threshold values (Cq) were normalized against GAPDH values then standardized to values obtained from mock treated cells. Standard error of the mean (SEM) was calculated from at least four biological replicates.

### Statistical analysis

Statistical analysis was performed with GraphPad Prism. An independent student′s t-test was used to compare the statistical significance of two groups, whereas two-way ANOVA was applied to compare more than two groups.

### Immunostimulation assays in human peripheral blood mononuclear cells (PBMCs)

Peripheral blood mononuclear cells (PBMCs) were obtained from healthy donors at City of Hope National Medical Center using discarded anonymous blood unit leukocyte filters (Pall). PBMCs were plated in 24-well plates (2.5 × 10^6^ cells/well) and transfected using DOTAP transfection reagent (Sigma-Aldrich) according to the manufacture′s protocol at a final RNA concentration of 80 nM. Cells were incubated at 37°C for 24 h. Supernatants from each well were collected, and IFN-α and IL-6 levels were quantified by enzyme-linked immunosorbent assay (ELISA) using 96-well ELISA plates coated with a human IFN-α or IL-6 antibody (Thermo Fisher Scientific), respectively. During this experiment, discarded peripheral blood from anonymous adult donors from the City of Hope Apheresis Center (Duarte, CA, USA) was used. The proposed research involved blood specimens from anonymous human subjects with no identifiers to age, race, ethnicity or gender. The information provided for the above submission was evaluated and determined not to involve human subjects research (45 CFR 46.102 (d)(f)). Therefore, it does not need to be approved nor does it need to undergo continuing review by the Institutional Review Board (IRB) in the City of Hope.

### RNA radiolabeling

RNA radiolabeling was performed following a published protocol (16). Briefly, the assay was performed by mixing oligonucleotide (100 pmol), T4 polynucleotide kinase (PNK) (2 μl from 10 U/μl), 10× PNK buffer (2 μl), [γ]-^32^P-ATP (2.5 μl ∼0.3 mCi, 6000 Ci/mmol), SUPERase-In (1 μl from 40 U/μl), and water in a 20 μl reaction. The mixture was then incubated at 37°C for 2.5 h and 5′-labeled RNA was extracted using phenol/chloroform. After the extraction, radiolabeled RNA strands were purified using standard denaturing gel procedures. To measure phosphorylation efficiency, equal amounts of radiolabeled single strand RNAs were resolved on a denaturing polyacrylamide gel, dried, imaged by phosphorimager, then bands quantified with ImageQuant.

### hAGO2 loading assay

The *in vitro* hAGO2 loading assay was performed essentially as described previously (16,17). Briefly, purified and 5′-radiolabeled siRNA strands were annealed and then gel purified by native polyacrylamide gel electrophoresis. Radiolabeled siRNA (5 × 10^5^ cpms) was rotated at room temperature for 1 h with 250 μl of cytoplasmic HeLa cell extract, 2.5 μl 100 mM ATP, 2.5 μl 1 M phosphocreatine and 2.5 μl creatine kinase (4 U/μl). The reaction was then rotated with 40 μl equilibrated Protein G Plus agarose (Pierce) and 1.5 μg of anti-hAGO2 antibody (Abcam) for 1 h at room temperature. Resin was washed then bound hAGO2 and loaded radiolabeled siRNA eluted by phenol-chloroform extraction and precipitation with 10 vol of 2% LiClO_4_ in acetone. The pellet was acetone washed, dried, then resolved on a 15% denaturing polyacrylamide gel. Finally, the gel was dried and RNA bands visualized by phosphorimager and quantified with ImageQuant software.

### Nuclease assays

Double stranded RNA samples were prepared in Tris-acetate buffer consisting of 45 mM Tris, 20 mM acetic acid and 7.6 mM MgCl_2_, pH 8 (pH adjusted using glacial acetic acid). Eighty pmoles of each duplex were annealed for 1 h from 95°C to 4°C, and then diluted with cell culture media (DMEM, 50% FBS, 5% AB/AM) to a final volume of 70 μl. Samples were incubated at 37°C, and aliquots (11.5 pmol) were taken at different time points and frozen until analysis. Aliquots were then loaded on a 15% denaturing polyacrylamide gel and stained with GelRed to visualize the bands.

The intensity of the bands from the gels were treated to extract quantitative data, i.e. the top band which represents the starting material (SM) and bottom band which represents the degradation products (DP). The ratio (Ratio1) which compares the amount of SM to the DP at each timepoint (*x*) was calculated by the following equation:

$$\begin{equation*}{\rm{Ratio}}1({{x}}) = {\rm{SM}}({{x}})/{{DP(x)}}\end{equation*}$$

Then, the value Ratio1 was normalized at each timepoint (*x*) with respect to the first timepoint (10 min) to compare the stability over time:

$$\begin{equation*}\% \,{\rm{Duplex}}\,{\rm{remaining}}({{x}}) = [{\rm Ratio1}(x)/{\rm{Ratio1}}(10)]\times 100\end{equation*}$$

### Molecular modeling studies

The hAGO2 protein and siRNA (17 bp) complex utilized for MD studies was obtained from the model reported in literature (18). The topology and coordinate inputs of protein and RNA complex were prepared using the xleap module in AMBER 14 (19). The protonation state of the amino acids was assigned using PDBPQR program at pH 7.0 in AMBER force field. The system was neutralized using KCl ions and excess ions (100mM) were added to mimic the physiological conditions. Mg^2+^ ions at the cleavage site were parameterized using a reported procedure (20,21). The partial charges for the 2′-5′-linked modifications were calculated at the nucleotide level using Gaussian 09 (HF/6-31G*) package (22). The calculated charges were then fitted using the RESP algorithm (23). The force field parameters for 2′-5′ linkage were derived from the previously reported crystal structure and MD studies (8). The force fields used for the RNA and the protein are bsc0_XOL3_ (24–27) and ff12SB (19), respectively. Using TIP3P water molecules, the system was solvated up to 8 Å from any of the solute atoms. The equilibration and MD simulations were performed as reported earlier (28,29). Unrestrained production MD simulations were performed for 250 ns using CUDA version of pmemd (30) in a GPU accelerated version (31,32) of AMBER 14 (19).

Particle mesh Ewald method was utilized for calculating the contributions from the non-bonded interactions with a cut-off of 10 Å. SHAKE was used to treat the bonds involving hydrogen atoms. The unrestrained MD simulations were performed in NPT ensemble of 2 fs time step. One atmospheric constant pressure was maintained using Bendersen weak-coupling barostat in a time constant of 1 ps (33). The MD simulations temperature (300 K) was maintained by Bendersen thermostat of 4 ps. MD trajectories were saved for every ps and then extracted at every 5 ps time interval for further analysis. Root mean square deviations (RMSDs) were calculated for the backbone heavy atoms of protein (CA, C and N) and RNA (P, O5′, C5′, C4′, C3′/C2′ and O3′) using CPPTRAJ module in AMBER 14 (34). The X3DNA package was utilized to compute the RNA helical parameters and backbone dihedral angles (35). Hydrogen bonds were considered based upon the heavy atom distance (donor-acceptor) cut-off of ≤3.3 Å and an angle cut-off of ≥135. The distance between the two heavy atoms were calculated using PTRAJ module. MD trajectories were visualized using UCSF Chimera (36). The free energy of siRNA–hAGO2 complex binding was computed using Molecular Mechanics/Poisson–Boltzmann Surface Area (MM/PBSA) method using an earlier report (37).

## RESULTS

### siRNA design and gene silencing assays

**Figure 2.**
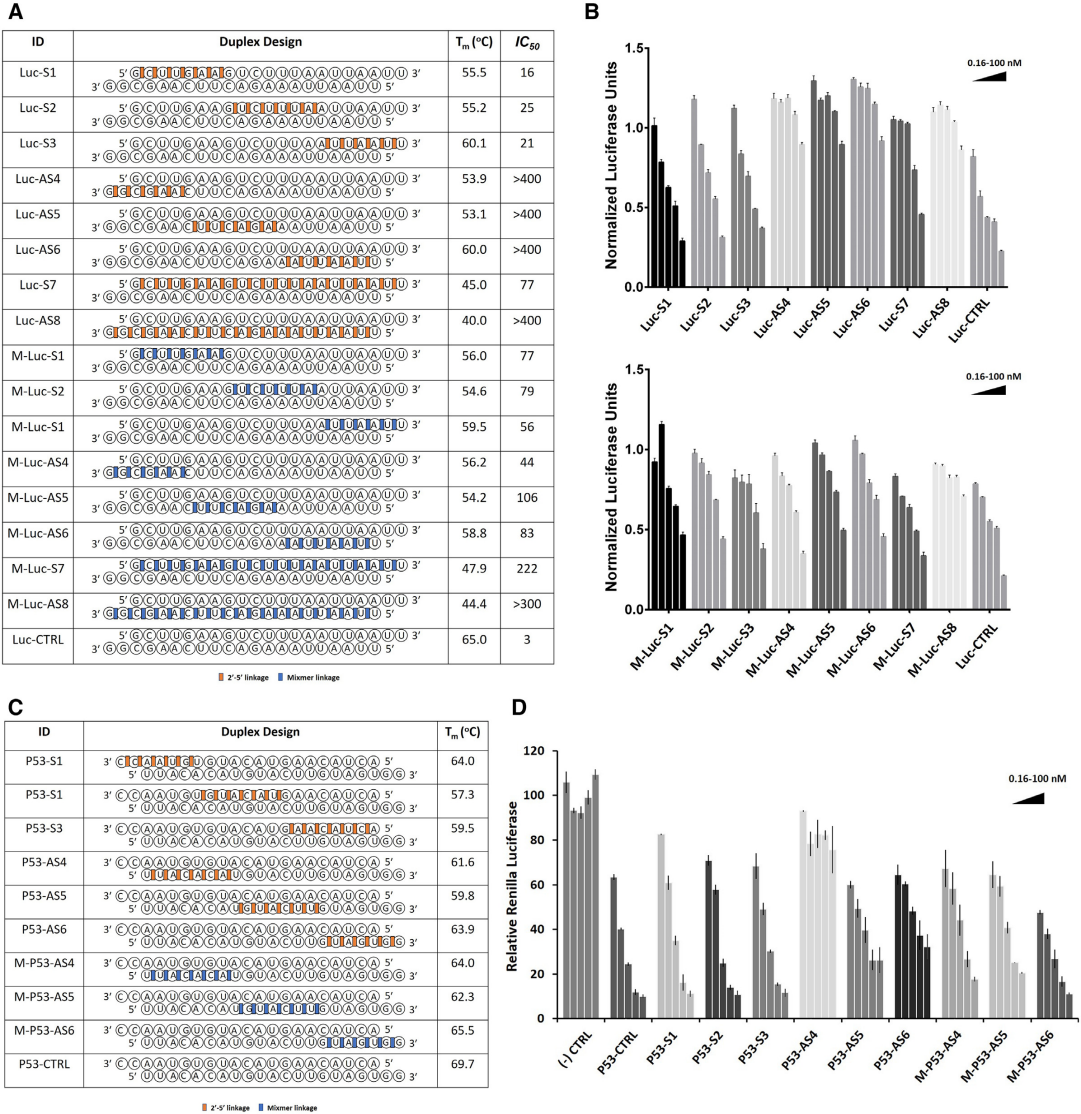
Thermal stability and gene silencing activity of sense (S) or antisense (AS) linkage-modified siRNAs targeting Firefly Luciferase (**A** and **B**) and P53 mRNAs (via a Renilla reporter) (**C** and **D**). Codes: 2′-5′ linkages (red), and mixed 2′-5′/3′-5′ linkages (blue); all other linkages are native 3′-5′ linkages. Error bars represent ±s.d. and *n* = 3 for all experiments.

We started our study by designing and synthesizing siRNA libraries targeting mRNA sequences of firefly luciferase and P53 mRNA containing 2′-5′ linkages at various positions in the sense (S) passenger or antisense (AS) guide strands (Figure 2). We started with a simple modification pattern by dividing each of the 21-nt sense and antisense strands into three sections each comprising a 7-nt segment (5′-end, central, 3′-end). Consecutive 2′-5′ linkages were placed on each of these segments, while keeping the rest unmodified (Figure 2A, C).

#### siRNAs targeting Firefly luciferase mRNA

siRNAs targeting Firefly luciferase mRNA were tested in HeLa cells (Figure 2A, B). Sense modified siRNAs (Luc S1, S2, S3) exhibited similar potency as the unmodified siRNA, and higher activity than a duplex with a fully sense modified strand (Luc-S7), indicating that a few 2′-5′ linkages in the sense strand can result in highly efficient mRNA knockdown. None of the siRNAs modified in the antisense strand (Luc-AS4, -AS5, -AS6, AS8) retained activity, consistent with previous findings (10).

As the modifications were located at the 5′-end of the antisense strand, we hypothesized that certain siRNAs may be inactive due to the inability of cellular kinases to install the 5′-phosphate on the antisense strand, required for RISC loading. To investigate this possibility, a selection of AS-modified siRNAs targeting firefly luciferase were chemically phosphorylated and screened against the luciferase mRNA. We found that 5′-phosphorylation did not rescue activity of the inactive siRNAs (siRNA-LucAS4, AS5, AS6, AS8, Figure 2A), suggesting that 5′-phosphorylation of the AS siRNA strand is unlikely to be the cause of reduced activity (Supplementary Figure S3).

#### siRNAs targeting P53 mRNA

To validate the results obtained above, we evaluated the impact of sequence variation on the activity of a second set of 2′-5′-linked siRNAs in which we targeted sites within the P53 mRNA (Figure 2C). Here, we followed the same modification pattern as above and used a vector that enabled monitoring of changes in expression of P53 fused to a Renilla reporter. Renilla luciferase levels were measured relative to the unmodified control reporter, following treatment with chemically modified siRNAs at a range of concentrations (Figure 2D). While siRNAs modified in different positions of the sense strand showed potencies comparable to the unmodified control, the siRNAs modified in the antisense strand either completely lost activity (P53-AS4) or showed diminished potencies (P53-AS5 and AS6). These results were in full agreement with the modified siRNAs targeting firefly luciferase. Similar to luciferase targeting siRNAs, no correlation was observed between activity and duplex thermal stability (Figure 2a, c).

### Properties of 2′-5′/3′-5-siRNA mixtures

Next, we tested the gene silencing activity of siRNA mixtures (‘mixmers’) varying in number and positioning of the 2′-5′ linkage. One motivation for doing this was to create modified RNA ‘pools’ that may have equal or better activity than our previously-tested structurally-fixed designs. Furthermore, by targeting mRNA with a statistical mixture of synthetic siRNA, we hoped to ‘dilute out’ innate immune responses of specific siRNA sequences. Finally, as indicated above, such mixtures have been hypothesized to have emerged as plausible prebiotic RNAs, and hence, we felt that studying their properties in a functional assay was of value.

The siRNA mixmers were readily prepared via solid-phase chemical synthesis by mixing and delivering equal amounts of ribonucleoside 2′P and 3′P amidites to the solid support during chain growth (see Methods section). The regioisomeric monomers were introduced at the previously defined segments of the S and AS strands (Figure 2a). This resulted in a mixed population of 21-nt oligomers, *all of the same sequence*, but containing a statistical mix of 2′,5′/3′,5′ linkages at each position. Thus, Mix-Luc S1, S2 and S3 all consist of a mixture of 2^6^ strands, whereas Mix-Luc AS4, AS5, and AS6 all consist of a mixture of 2^20^ strands. Appropriate amounts of these sense strand mixtures were complexed with RNA antisense strands and tested for knockdown potency in our *in vitro* luciferase assays.

siRNAs with ‘mixed’ 2′-5′/3′-5′-AS strands (M-Luc-AS4, AS5, AS6) displayed significantly higher knockdown potencies relative to siRNAs with 2′-5′-modified AS strands (Luc-AS4, -AS5, -AS6). Again, we observed that siRNAs with mixed 2′-5′/3′-5′-S strand modifications performed better relative to siRNAs with mixed 2′-5′/3′-5′-AS strands. Some of the siRNAs with mixed 2′-5′/3′-5′-S strands exhibited similar activity to the unmodified siRNA (Figure 2A, B). A similar pattern was observed for the siRNAs targeting P53 mRNAs. Here, siRNAs with a mixed 2′-5′/3′-5′-AS strand performed similar (M-P53-AS5) or better (M-P53-AS4, AS6) compared to siRNAs with consecutive 2′-5′-linkages (Figure 2C, D).

Thermal denaturation curves of mixmer siRNA duplexes exhibited sigmoidal melting profile, with similar or higher *T*_m_ values, in comparison with their 2′-5′ modified counterparts (Figure 2A, C). Stability differences were more pronounced for siRNAs containing a *fully modified* S or AS strand. Circular dichroism (CD) spectral profiles of a selected number of 2′-5′- and mixmer-modified siRNAs were all characteristic of A-form right-handed helices and were similar to spectrum of the unmodified RNA (Supplementary Figure S2).

### Positional screening of 2′-5′ linkage in siRNAs with modified AS strand

**Figure 3.**
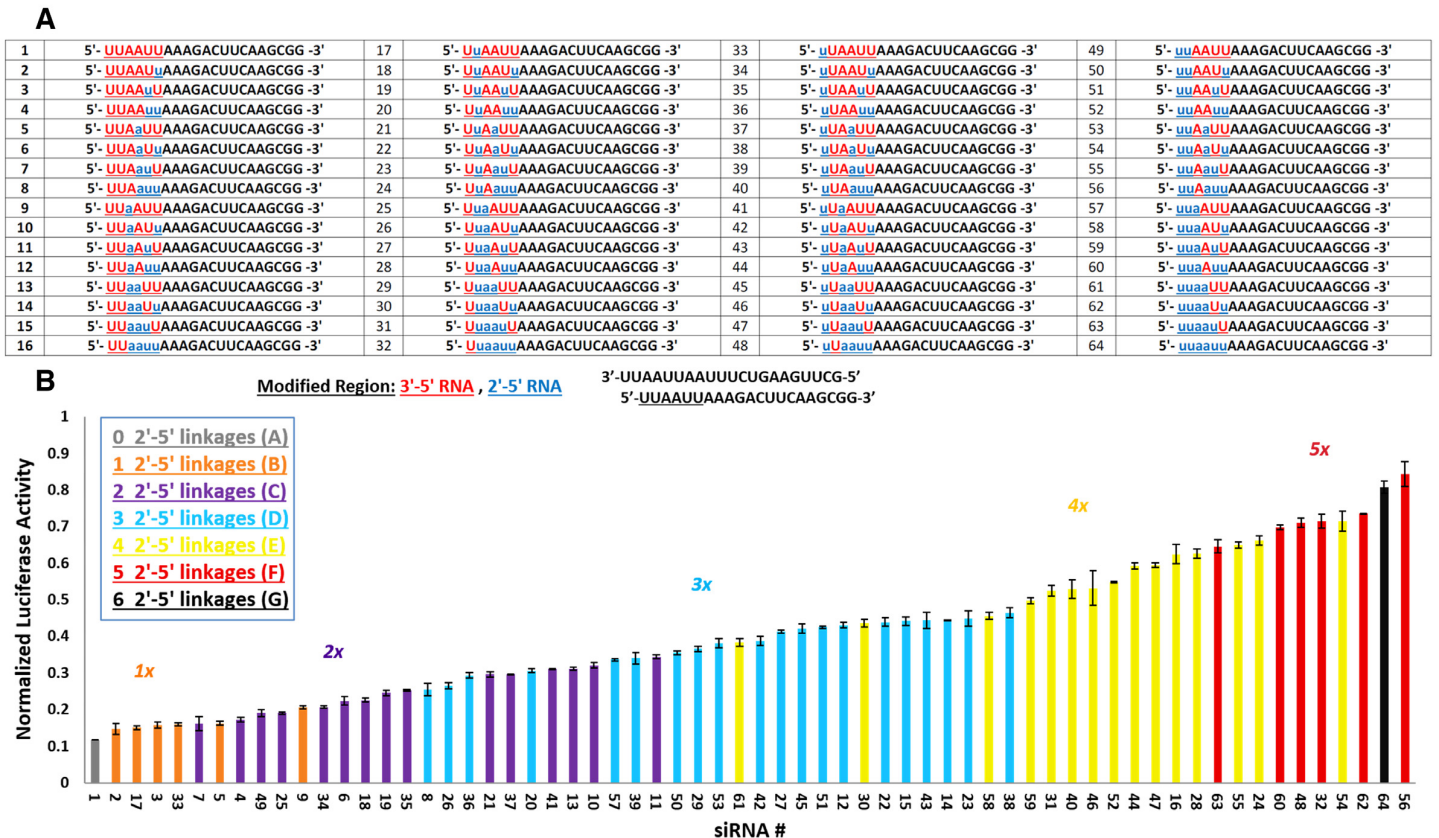
Positional screening of 2′-5′ linked siRNAs targeting firefly luciferase in a library of 64 siRNAs. (**A**) siRNAs modified at the six nucleotides on the 5′-termini of guide strands are represented by the number corresponding to their guide strand listed in the table. (**B**) Bar graph represents the gene silencing activity of the modified siRNA library targeting Firefly luciferase and is sorted based on siRNA activity. Bars of the same color represent siRNAs with the same number of linkage modifications. Error bars indicate standard deviation and *n* = 3 for all experiments.

To assess whether single and/or multiple 2′-5′-substitutions are position dependent, we next focused on a library of siRNAs targeting firefly luciferase containing all possible 2′-5′ substitutions within the seed region, which is known to be very sensitive to chemical modifications (38,39). Thus, all possible modification patterns were introduced in the UUAAUU segment generating a library of (2)^6^ = 64 antisense strands (Figure 3). Each member of the library was then annealed to the complementary sense strand for luciferase assay screening.

Gene silencing experiments were performed at one siRNA concentration (20 nM) and their ability to knock-down the firefly luciferase mRNA was measured. siRNAs having the same number of 2′-5′ linkages are shown as color-coded subsets (Figure 3). The activities of each library member are arranged from the most active (#1) to the least active siRNAs (#56 and #64). As expected, the most active siRNA among all members was the unmodified siRNA, however, the singly modified siRNAs (i.e. 2′-5′ linkage at positions 1, 2, 4 and 5 of the seed region) retained comparable activities (Figure 3A, duplexes #2, #3, #17 and #33). Additional findings were: (i) siRNA activity generally decreases with increasing number of 2′-5′ linkages. A few exceptions were noted. For instance, siRNA #61 with four modifications is much more active than many of the siRNAs with three modifications, suggesting that *modification pattern* also plays a role in siRNA activity; (ii) within each subset, *the most active have a consecutive arrangement of* 2′*-*5′ *linkages*, whereas the least active have alternating 2′-5′ and 3′-5′ linkages, e.g. siRNAs #7 versus #11 (2 modifications subset), #8 versus #38 (three modifications subset), and #61 versus #54 (four modifications subset).

### siRNAs targeting and endogenous gene target (DBR1)

**Figure 4.**
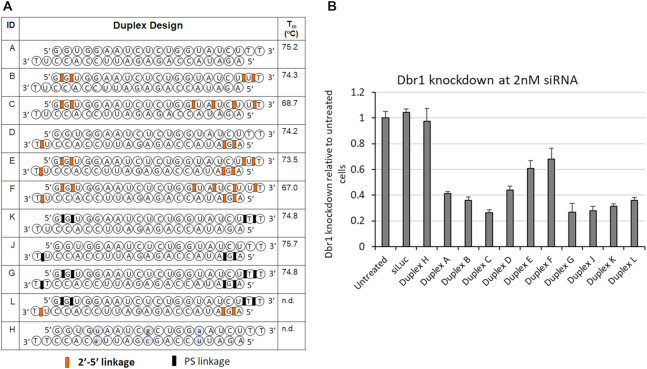
(**A**) Sequence, 2′-5′ modification pattern, thermal stability, and activity of siRNAs targeting Dbr1 mRNA. Duplex H (with three base pair changes indicated in small cap letters) and duplex siLuc (targets luciferase mRNA) represent negative controls. (**B**) Knockdown of Dbr1 mRNA levels with native, modified, and control siRNAs duplexes shown in panel A. Error bars are standard error of the mean of four or more replicates.

To examine the applicability of our 2′-5′/3′-5′-siRNA designs to yet another target, we next prepared and tested several new siRNAs targeting Dbr1 mRNA (Figure 4A). Dbr1 is the key RNA lariat debranching enzyme in mammalian cells. It is central to RNA metabolism because its activity is required for intron turnover and to produce small nucleolar RNAs and microRNAs encoded in intronic RNA. However, its knock-down is usually not detrimental to cells in the short term. We saw little or no toxicity when performing siRNA treatments up to 20 nM in HeLa cells. We selected a concentration of 2 nM siRNA to compare knockdown across several designs (Figure 4B).

2′-5′-Linkages were introduced at the 5′/3′-termini of the AS/S strands to potentially enhance nuclease activity (e.g. duplexes B, D and E, Figure 4). By placing alternating 2′-5′/3′-5′-steps at the 3′-end of the sense strand, we hoped to destabilize the duplex at the 5′-end of the antisense strand and thus, favor loading of the antisense strand into RISC (duplexes C and F, Figure 4) (40,41). As a comparison, the corresponding phosphorothioate (PS) modified duplexes (K, J, G) were prepared and tested.

Dbr1 mRNA levels were determined by RT-qPCR following treatment with siRNAs at 2 nM (Figure 4B). All sense modified siRNAs (duplexes B, C and K) were of greater potency than the unmodified siRNA, with C being the most notable one in terms of activity enhancement in this experiment. siRNAs with modified AS and S strands (E, F) had lower activity relative to the native or PS-modified duplex (G, Figure 4B). Generally, the results fit well with results obtained for the siRNAs that target luciferase: 2′-5′ linkages are better tolerated in the sense strand. Of interest, a chimeric duplex constructed from a 2′-5′/3′-5′ sense strand and a PO/PS-modified antisense strand (duplex L) was as potent as the unmodified siRNA (duplex A).

### Nuclease stability assays

**Figure 5.**
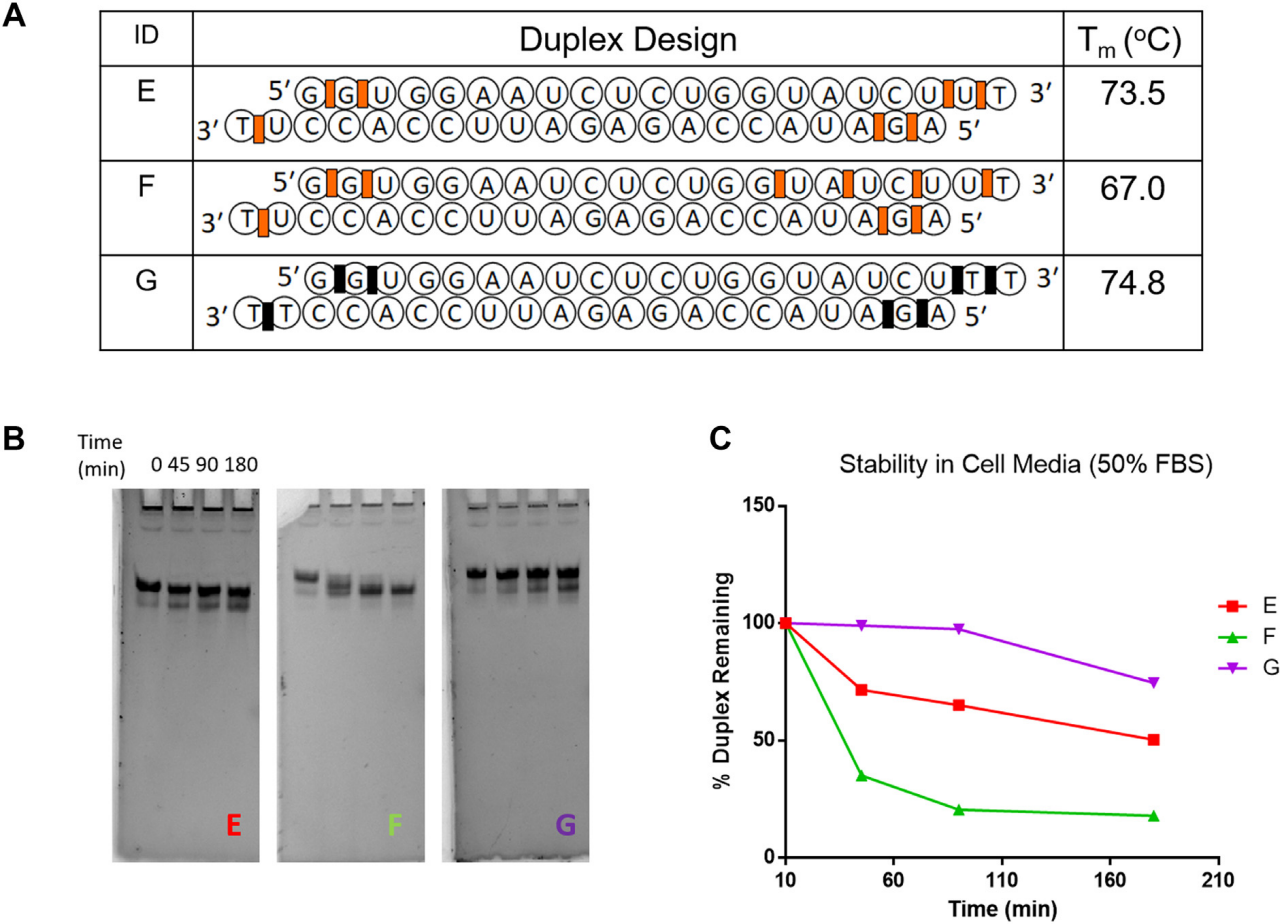
Serum Stability of various RNA duplexes in 50% FBS and cell culture media (DMEM). (**A**) Sequences of the duplexes tested. (**B**) 15% Denaturing PAGE gels of the duplexes at various timepoints. (**C**) Plot of the % duplex remaining over time. Codes: 3′-5′-phosphorothioate linkages (black rectangles; duplex G), 2′-5′ linkages (red rectangles; duplexes E and F), all other residues are linked via 3′-5′ linkages.

We tested the stability of the dsRNAs with different modifications by incubating in serum-rich cell culture media, which is composed of 50% fetal bovine serum (FBS) in Dulbecco's modified Eagle's medium (DMEM) (Figure 5) (42,43) and calf spleen phosphodiesterase (SPDE, Supplemental Material). Aliquots were taken at hourly time points and analyzed by denaturing polyacrylamide gel electrophoresis to assess the effects of the chemical modifications on nuclease sensitivity (Figure 5 and Supplementary Figure S4). In the FBS media, all duplexes convert to a faster moving band which we ascribe to a blunt-ended duplex resulting from cleavage of 3′-overhangs (dT(PS)dT and rU(2′-5′)dT). The data show that among the three duplexes shown in Figure 5 and Supplementary Figure S4, the PS modified dsRNA (G) was the most stable. When more 2′-5′ linkages were introduced in the sense strand nuclease stability dropped significantly (compare duplex E versus F). We hypothesize this is due, at least in part, to the increased terminal fraying expected for duplex F compared to duplex E. The lower overall *T*_m_ of duplex F relative to duplex E supports this notion (Figure 5). The nuclease stability of the corresponding unmodified duplex A was not determined under the same conditions (50% FBS). However, preliminary assays in 10% FBS media showed that duplex A (dTT overhangs) and other native siRNAs of similar sequence are more resistant to hydrolysis relative to the 2′-5′-modified duplex E (Supplemental Information, Figure S4). While unexpected, this finding parallels that of Usher and McHale that 2′-5′ linkages are more prone to hydrolytic cleavage relative to natural 3′-5′ linkages, and more so when the 2′-5′ linkages are embedded in a helical (duplex) structure (44). The apparent higher susceptibility of 2′-5′-PO (duplex E) versus 3′-5′-PS linkages (duplex G), may explain at least in part the higher silencing activity of the latter (Figure 4). In light of these results, it would be interesting to assess the nuclease and gene silencing activity of siRNA duplexes containing 2′-5′-phosphorothioate (PS) linkages.

### Incorporation of 2′-5′-linkages abrogates immune-stimulatory activity of siRNAs

**Figure 6.**
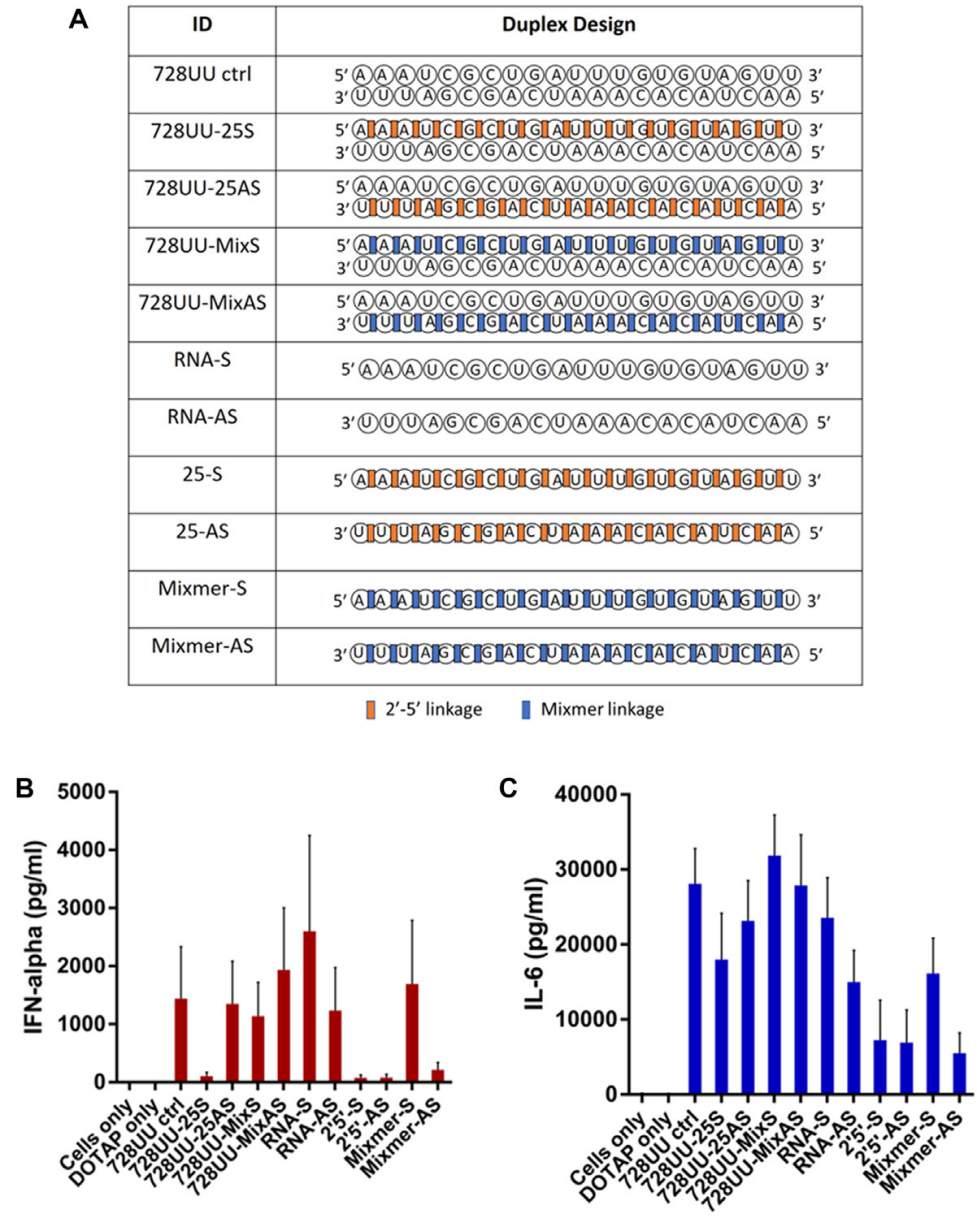
Comparison of immune-stimulation in PBMCs after treatment with the linkage-modified and unmodified siRNA 728UU (**A**). IFN levels were measured 24 hours after siRNAs were transfected in duplicate using DOTAP. IFN-α levels (**B**) and IL-6 levels (**C**) in response to treatment with unmodified control siRNA (728UU ctrl) and its constituent single strands (RNA-S and RNA-AS); 2′-5′-modified siRNAs (728UU-25S and 728UU-25AS) and their constituent single strands (2′-5′S and 2′-5′-AS); mixmer siRNAs (728UU-MixS and 728UU-MixAS) and their constituent mixmer single strands (Mixmer S and Mixmer AS). Cells only was the negative control with no reagents. DOTAP only treatment was transfection without siRNA. Data were collected in duplicate for each of six donors. Bars indicate standard deviation.

Chemical modification has proven particularly useful for reducing immune-stimulatory activity of certain siRNA sequences. For instance, treatment of human peripherial blood mononuclear cells (PBMCs) with siRNA can elicit strong interferon-α (IFN-α) responses (45,46). To investigate whether this also applies to 2′-5′ modified siRNAs, we constructed a control siRNA duplex with reported strong immune-stimulatory effects arising from an internal UGUGU motif present in the sense strand (45,46). PBMCs were treated with DOTAP formulated modified and unmodified duplexes and their constituent single strands (Figure 6A) (14). The immune-stimulatory effects were assessed by measuring IFN-α and IL6 levels using ELISA.

PBMCs treated with the unmodified siRNA (728UU ctrl) and its constituent unmodified S and AS single strands (RNA-S and RNA-AS) led to a significant increase in IFN-α production compared to the mock treatments (i.e. cells with either no treatment or cells exposed only to the transfecting agent, DOTAP) (Figure 6B, C). Similarly, introduction of 2′-5′ linkages in the AS strand of the duplexes (i.e. 728UU-25AS and 728UU-MixAS) had little or no effect on IFN-α production. In sharp contrast, treatment with siRNA 728UU-25S (duplex with a 2′-5′-linked S strand and unmodified AS strand) reduced IFN-α levels to nearly undetectable levels. The same was true for the 2′-5′-linked single strands (2′-5′-S and 2′-5′-AS) and the mixmer AS single strand (Mix-AS). Both mixmer duplex 728UU-mixS and mixmer S strand induced IFN-α and IL-6 secretions to a significant extent. Induction of IL-6 in all cases followed the same general trend, although reduction in IL6 levels upon treatment was not as pronounced (Figure 6c). These results suggest that complete replacement of 3′-5′ linkages with 2′-5′ linkages abrogates the immune-stimulatory profile of single-stranded oligonucleotides.

### Molecular modelling and dynamic studies on 2′-5′-modified siRNAs: hAGO2 complexes

With a few notable exceptions (38,47–50), chemical modifications in siRNAs have been reported to diminish RNAi activity, particularly when placed at the seed region of the AS strand (nucleotides 2–8 from 5′ terminus) (51–53). Thus, it was reasonable to hypothesize that certain patterns of 2′-5′/3′-5′ linkages likely interfered with essential hAGO2 interactions.

**Figure 7.**
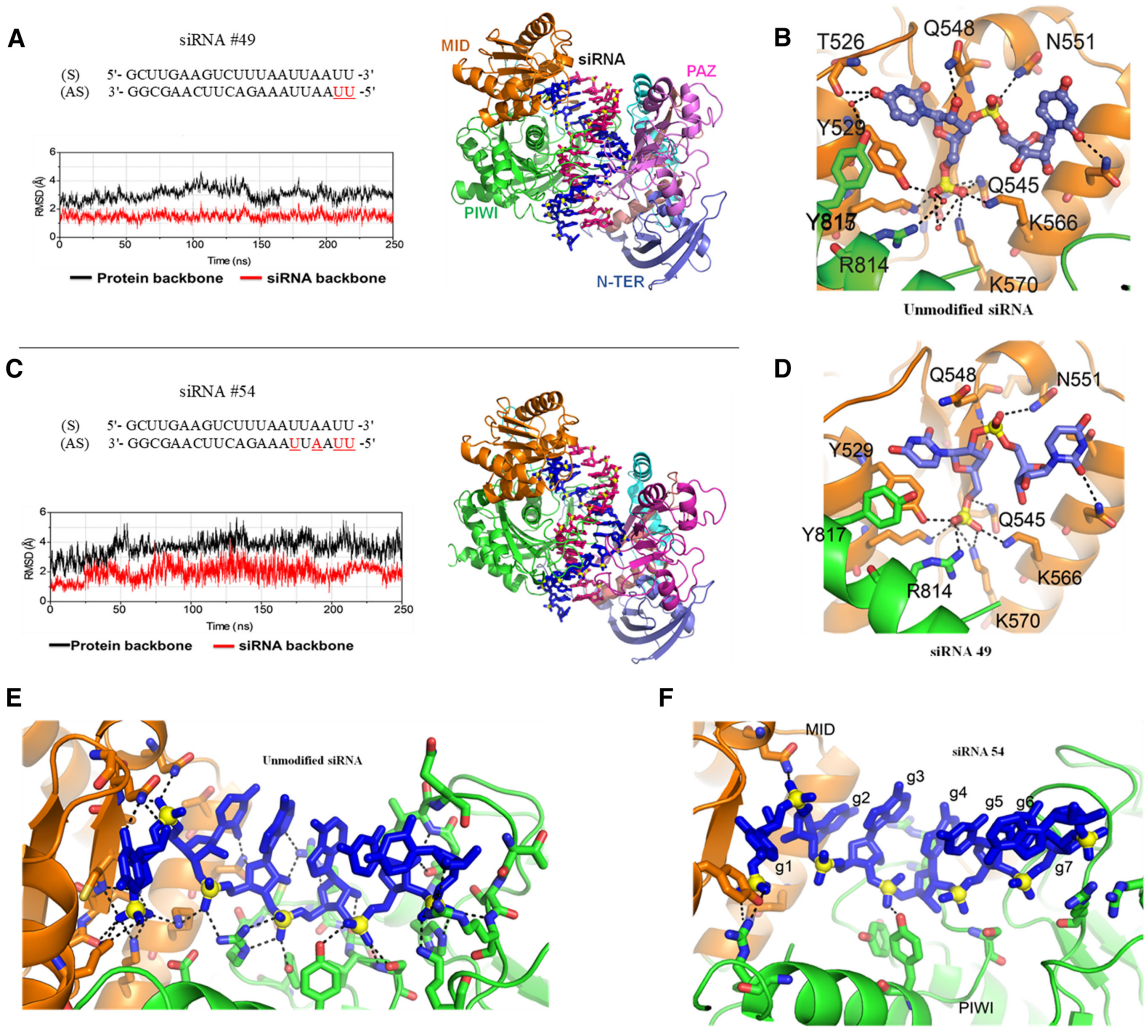
MD snapshots averaged from the last 50 ns of the MD simulations and root-mean-square deviation (RMSD) graphs from the MD simulations of the 2′-5′ linked siRNAs #49 (**A**) and #54 (**B**) and hAGO2. hAGO2 is presented in cartoon, each domain of the protein is labelled in a different colour; siRNA backbone is presented in ball and stick with AS strand in magenta and passenger strand in blue. RMSD graphs were calculated over 250 ns using equilibrated structures as the reference with hAGO2 displayed in black and siRNA displayed in red. Non-covalent interactions between hAGO2 and 5′ terminal of unmodified siRNA (**C**), (**E**); siRNA #49 (**D**) and siRNA #54 (**F**) are displayed. Key non-covalent interactions between hAGO2 and siRNAs are labelled in black dashed line. Legend: 3′-5′ linked nucleotides (black), 2′-5′ linked nucleotides (red).

To test this hypothesis, we carried out MD studies on unmodified and modified siRNAs bound to an open conformation of hAGO2. The hAGO2 protein complexed to siRNA (nucleotides 2–19 are base paired) was adapted from a model reported in literature (details in experimental section) (18). The final MD snapshot of the siRNAs and hAGO2 after 250 ns of MD simulation is shown in Figure 7. The RMSD graph of the siRNA #49/hAGO2 complex (Figure 7A) shows that the complex was quite stable during the simulations. However, siRNA #54/hAGO2 complex, as shown in the corresponding RMSD graph (Figure 7B), was found to be highly flexible, especially around the 2′-5′ modified region.

Next, interactions of hAGO2 with siRNA #49 (active) and siRNA #54 (inactive) were assessed by molecular dynamics (MD) simulations and binding energy calculations. Comparison of the averaged structures from the last 50 ns of the 250 ns MD simulations revealed that siRNA #49 only lost a very few non-covalent interactions (Q548, T526, and Y529) at the 5′-binding pocket of hAGO2 (Figure 7C and E). In contrast, many non-covalent interactions between the protein and the antisense strand of siRNA #54 are lost in the course of MD simulations (i.e. Q545, K570, K566, N551, K550, R792, R795, H753, Q757 and Y790) (Figure 7D and F). These findings are further supported by the binding free energy calculations (Supplementary Table S4), which indicates that the noncovalent interactions responsible for the formation of siRNA #49-hAGO2 complex was not significantly affected in comparison to those in the unmodified complex (ΔΔ*G* = 38 ± 9). In contrary, the large variation in the ΔΔG values for the siRNA #54-hAGO2 complex (ΔΔ*G* = 124 ± 9) shows that the non-covalent interactions were significantly affected due to extensive presence of 2′-5′ linkages in siRNA #54. Overall, these factors likely account for the loss in activity observed for siRNA #54 relative to siRNA #49.

#### 2′,5′-RNA can form a ‘clover leaf’ bend critical for siRNA activity

The 5′ nucleotide in siRNAs has a crucial bend in the backbone referred to as the ‘clover leaf’ junction (Supplementary Figure S5). Deviation from this structure due to modification can be linked to loss of non-covalent interactions with hAGO2 (54,55). Torsional angles for siRNAs #49 and #54 varied significantly at the site of modification relative to the native siRNA (Supplementary Table S2). However, despite losing interactions with three amino acid residues, the ‘clover leaf’ bend at g1-g2 in both siRNAs #49 and #54 was maintained (Figure 7E, F and Supplementary Figure S5).

#### The effect of 2′-5′ linkage on sugar conformation and helical structure

**Figure 8.**
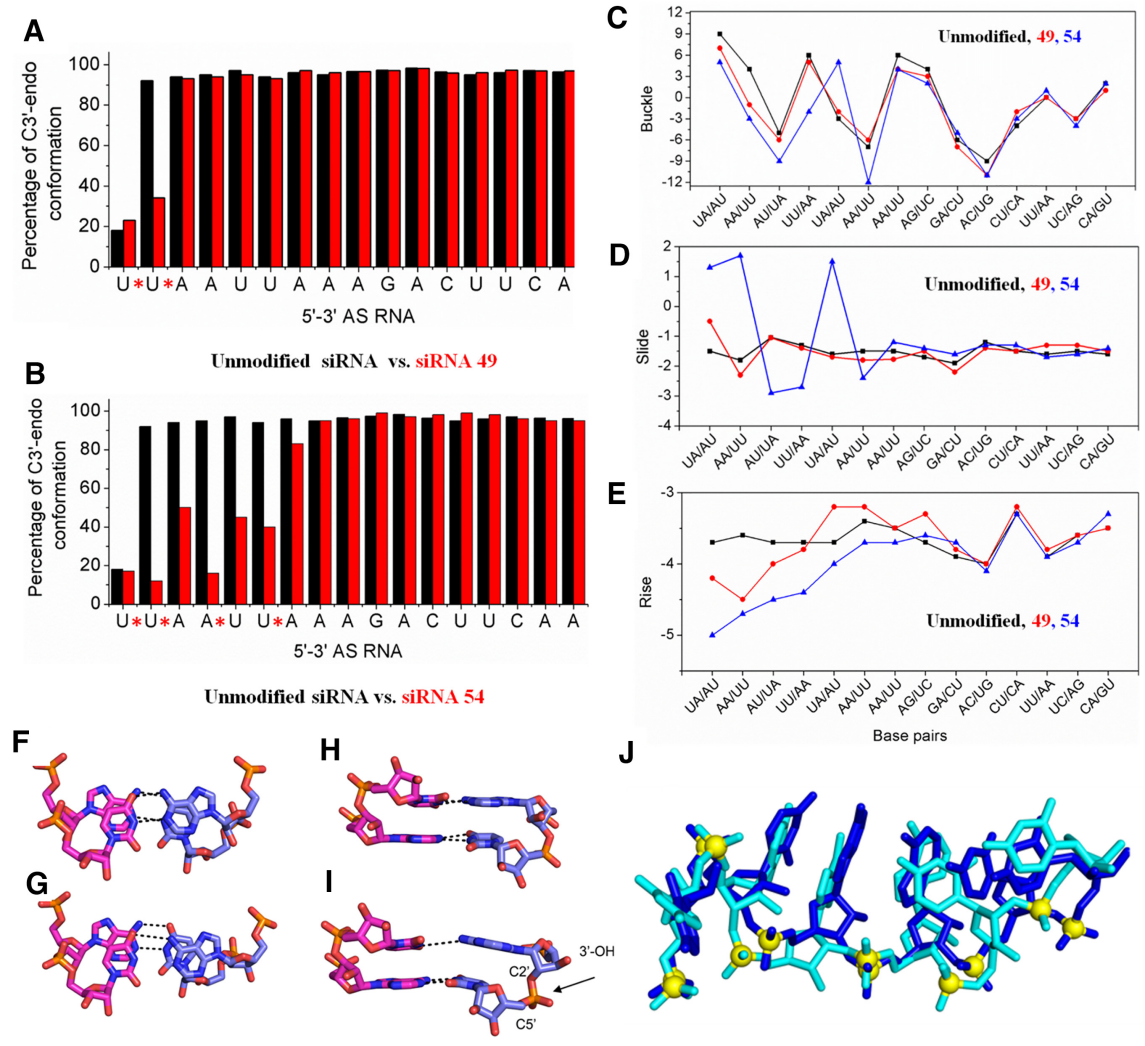
Sugar conformation of nucleotides in unmodified (black bars) versus 2′-5′ modified (red bars) siRNAs #49 (**A**) and #54 (**B**). * indicates a 2′-5′ linkage. Base pair step parameters (buckle (**C**), slide (**D**) and rise (**E**)) of the unmodified and modified siRNA duplexes from the averaged structure obtained from the last 50 ns of the 250 ns MD simulations. (F–I) MD snapshot depicting the base pair and base stacking architecture of g4–g5 step in unmodified and modified siRNA 54. Base pairing of the (**F**) unmodified and (**G**) modified siRNA duplex. Base stacking of the successive base pair steps in the (**H**) unmodified and (**I**) modified siRNA duplex. The black dashed lines indicate the WC H-bond between the bases. The modified linkage is highlighted using an arrow. (**J**) Superimposed snapshot of unmodified (blue) and modified siRNA 54 (cyan) AS strands (sense strand is not shown for clarity), illustrating the deviations in the backbone conformation and suboptimal intra-strand base stacking. RNA is represented as sticks and the phosphorus atoms are shown as yellow spheres.

The conformation of sugar rings was calculated from the 250 ns MD trajectory. Except for the nucleotide at position 2 (g2), the sugar rings of unmodified siRNA and siRNA #49 adopted the same conformation, i.e. C2′-*endo* at g1, and C3′-*endo* at g3-g17, perhaps not surprising given that siRNA #49 is modified only at g1 and g2 (Figure 8A). In siRNA #54 (2′-5′ linkages at g1, g2, g4 and g6) the linkage modification shifted the sugar puckers from C3′-*endo* to C2′-*endo* forms both in the 2′-5′ linked nucleotides, as well as their adjacent 3′-5′ linked nucleotides (Figure 8B). While such heterogeneous sugar-backbone conformation likely maintains the overall A-form duplex structure (8), it is predicted to affect key siRNA-hAGO2 interactions as shown above.

Structural parameters such as rise, buckle and slide are often used to define the orientation and position of each base pair with another and to show the stacking geometry between neighboring base pairs in an RNA double helix (56,57). Our calculations showed that extensive modification, as in siRNA-54, result in significantly deviated buckle, slide and rise parameters in comparison with those in the unmodified siRNA (Figure 8c, d, e). In agreement with previous reports (8), our calculations suggest that the increase in the buckle amplitude upon 2′-5′ modification results in lower base overlap and reduced intra-strand stacking (Supplementary Table S3), thereby destabilizing the duplex (Figure 8F–J). Hence, we conclude that differences in phosphate orientation, variations in sugar conformation, loss of W–C H-bonds (Supplementary Table S3), and slight local deviation in A-form geometry are collectively responsible for the poor RNAi activity of siRNA-54 and likely other inactive 2′-5′-modified siRNAs.

### hAGO2 loading assay

**Figure 9.**
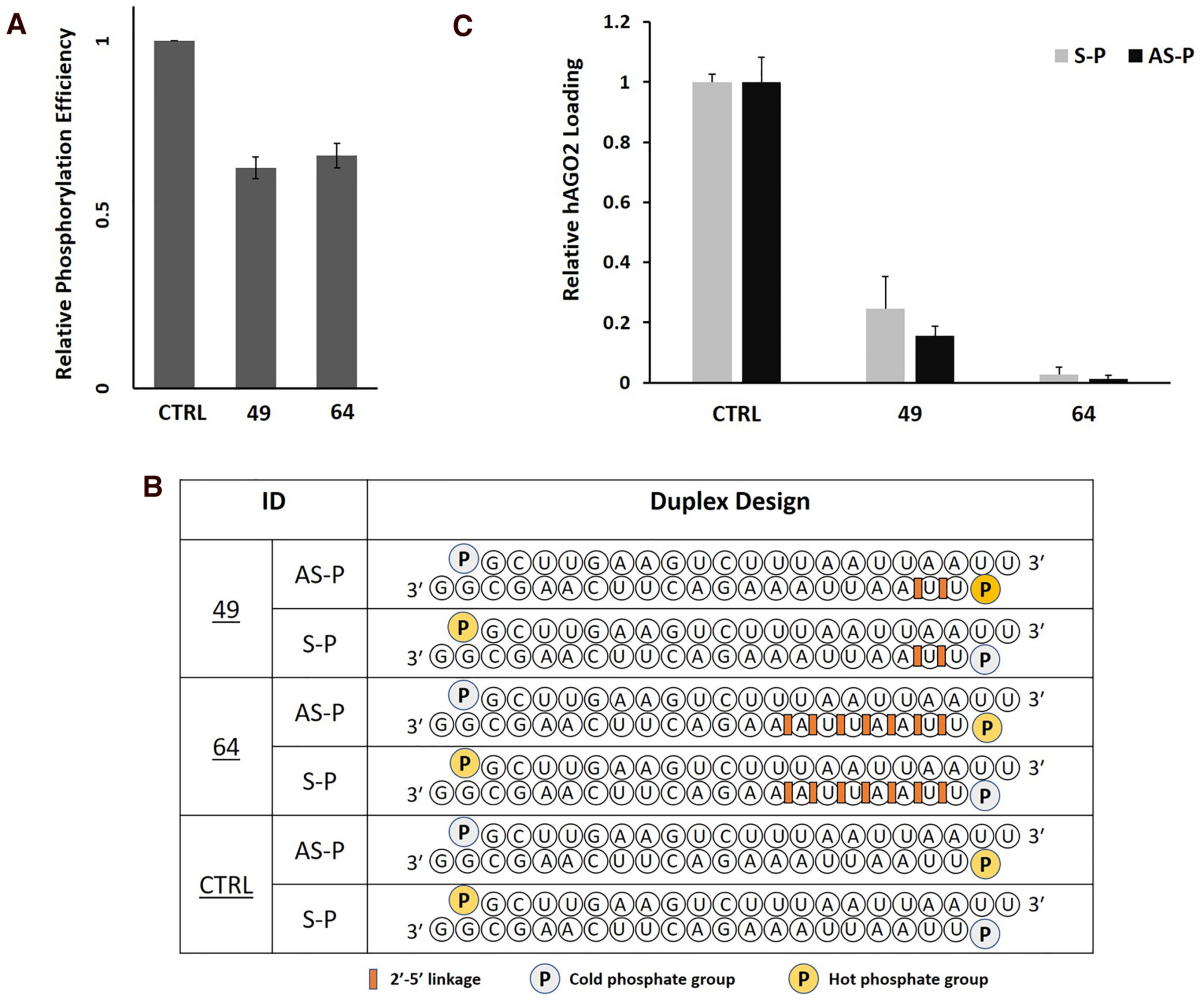
(**A**) Kinase efficiency in radiolabeling unmodified (CTRL-AS) versus 2′-5′ linked siRNA strands (49-AS and 64-AS). (**B**) Unmodified and linkage modified siRNAs (#64 and #49) radiolabeled on S and AS strands that were used in AGO2 loading assay (**C**). Error bars represent ±s.d. and *n* = 3 for all experiments.

To further test the possibility that efficiency of siRNA loading into hAGO2 was compromised by extensive 2′-5′-modification, a hAGO2 loading assay was employed. The native siRNA and 2′-5′-modified siRNAs #49 and #64 were chosen as model duplexes in this study. We first tested phosphorylation efficiency in vitro. In this assay, T4 polynucleotide kinase adds a radioactive phosphate group from [γ-^32^P] ATP to the 5′-OH of the oligonucleotide strand. Quantification of the amount of radioactivity incorporated into an RNA substrate after resolution on a gel allows for comparison of substrate compatibility with the kinase. This assay revealed that siRNA modified at the 5′ termini of AS strand with two and six 2′-5′ linkages (AS-49 and AS-64) were efficiently phosphorylated by the kinase (63% and 67% phosphorylation efficiency, respectively; Figure 9A). These findings confirmed our previous findings that poor 5′-phosphorylation of 2′-5′-modified siRNAs was not the cause for the reduced activity in cells. Next, unmodified siRNA and siRNA #49 and #64 were radiolabeled at the 5′ termini of their S or AS strands (Figure 9b) and the resulting duplexes incubated with HeLa cell cytoplasmic extracts. Using an anti-hAGO2 antibody, hAGO2 was immunoprecipitated and radiolabeled siRNA strands that co-purified were resolved on a gel for quantification (16,17). As expected from siRNA design principles, we observed a strong bias for loading the antisense strand over the sense strand (Supplementary Figure S6). However, loading of siRNA #49, both for antisense and sense strands, was reduced down to ∼20% (Figure 9C). Loading of siRNA #64 was negligible. Thus, poor loading may explain the lack of activity of certain 2′-5′-modified siRNAs, such as siRNA #64. We expected higher loading activity for siRNA #49, given its efficient knockdown of Luciferase in cells. These results suggest that additional factors beyond hAGO2 binding to siRNAs play important roles in the final observed siRNA activity. These factors could include the involvement of other loading factors (58), or the likelihood that only a subset of hAGO2 needs to be loaded with siRNA to achieve catalytic decay of target mRNAs (59).

## DISCUSSION

In recent years nucleic acids have emerged as extremely promising candidates for drug therapy in a wide range of diseases. They are now recognized as the third major drug discovery platform in addition to small molecules and antibodies. With an ability to engage targets that are otherwise undruggable by conventional therapeutics, nucleic acid-based drugs have opened new avenues for treating intractable diseases (60). RNA interference (RNAi) is a natural small RNA-guided mechanism that can control gene expression in mammalian cells. Creation of chemically modified synthetic small interfering RNAs (siRNAs) to guide RNAi has made therapeutic control of gene expression possible. Recent progress has been rapid, broad, and exciting, with two siRNA-based drugs on the market and dozens of candidates in early to late-stage clinical trials (60–62).

Our motivation to study 2′-5′/3′-5′-siRNA duplexes arose in part from studies demonstrating that incorporating several 2′-5′-linkages to an otherwise unmodified RNA duplex do not cause significant structural perturbations (8,9). Therefore, we hypothesized that combining 2′-5′ and 3′-5′-linkages would produce modified A-form siRNA structures similar to the native siRNA substrates recognized by RISC. While it is known that an increased number of 2′-5′-linkages decreases RNA duplex stability in a linear fashion, destabilization is dependent on the positions as well as the arrangement of these 2′-5′-linkages within the duplex (6). Therefore, thermodynamically stable 2′-5′/3′-5′-RNA duplexes can be readily obtained for structural and functional studies (5–10).

To this end, we chose to investigate siRNAs composed of 2′-5′ and 3′-5′-linkages (Figure 1). Screening of several siRNAs revealed which regions of the sense or antisense strand are sensitive to modification by 2′-5′-linkages. The 2′-5′ linkage was well tolerated in the sense strand of siRNAs (Luc, p53 and Dbr1 siRNAs, Figures 1, 2 and 4). However, it effectively abolished activity when introduced in ‘blocks’ within the antisense strand (Figure 2). Provided there were not too many, 2′-5′ linkages were tolerated at the 5′-terminus and certain positions of the seed region of the antisense strand, which is known to be particularly sensitive to chemical modifications (Figure 3) (38,39).

To further assess the impact of 2′-5′ linkages on gene silencing activity, we prepared a 64-member library containing all possible 2′-5′/3′-5′-permutations within the AS seed region. Our findings revealed that with a few exceptions, activity generally decreased with an increasing number of 2′-5′ linkages. Furthermore, the *modification pattern* appeared to be important, with the more active siRNAs containing a few contiguous 2′-5′ linkages relative to siRNAs with alternating 2′-5′ and 3′-5′ linkages (Figure 3). Our modeling experiments suggested that the reduced activity of heavily modified siRNAs is related to *local* structural changes induced by certain 2′-5′/3′-5′ patterns affecting interactions with hAGO2. Consistent with these results, hAGO2/siRNA loading experiments suggest that the lack of activity of heavily modified 2′-5′-modified siRNAs is due, at least in part, to reduced binding to hAGO2.

In an attempt to reduce the number of siRNAs in these screens, and possibly generate more active siRNAs, we tested mixtures of 2′-5′-/3′-5′-siRNAs incorporating modifications within the S and AS strands. These siRNAs were readily prepared via solid-phase synthesis by mixing and delivering equal amounts of ribonucleoside 2′P and 3′P amidites to the solid support during synthesis, resulting in a mixed population of RNA *regioisomers* with the same base sequence. In some ways, this strategy parallels the stereoisomeric effect of phosphorothioates (PS) by introducing sulfur at the phosphate non-bridging positions in both the S and R configurations, creating a mixture of *diastereoisomers* in this case. The difference is that the PS isomers are produced during the coupling and sulfurization steps, whereas in our approach, the 2′P and 3′P monomers are pre-mixed and delivered to the solid support during RNA chain assembly. Thus, the individual molecules possess randomly incorporated 2′-5′ and 3′-5′ linkages and so are distinct from the chemically-defined oligos obtained from selective incorporation of 2′-5′ or 3′-5′ linkages at determined positions.

We found that siRNAs with ‘mixed’ 2′-5′/3′-5′-AS strands displayed significantly higher knockdown potencies relative to siRNAs with 2′-5′-modified AS strands. While this can be attributed to the presence of ca. 60% 3′-5′-linkages in the duplex (see Materials and Methods), it is conceivable that some of the individual siRNA molecules of the library exhibit higher activity relative to the unmodified siRNA duplex. In fact, we have yet to explore the impact of *site-specific* incorporation of 2′-5′-linkages outside the seed and 3′-terminus of the antisense strand. The central region (cleavage site/Ago 2 PIWI domain), is also critical in the design of chemically modified siRNAs for therapeutic use, where unique structural features and RNA-protein interactions exist.

An important finding of our studies is that siRNAs tolerated one or two 2′-5′-linkages at (or near) the 5′ terminus of the antisense strand (Duplexes #33 and #49, Figure 3; and duplex D, Figure 4). Our modeling experiments provided a possible rationale for this observation: both the native as well as the 5′-rN_1_(2′-5′)rN_2_ dinucleotide adopted the ‘clover leaf’ bend and sugar pucker (C2′-*endo*) that are critical for anchoring the 5′-phosphate to AGO2 MID domain. Given the activity observed for some of our 2′-5′-/3′-5′-siRNAs (e.g. duplexes D and L, Figure 4), the 2′-5′-modification may offer an alternative to 5′ and 3′ PS modification currently used in the clinic for terminal stabilization. While the nuclease resistance of 3′-5′-(PO or PS) linkages was best, it remains to be seen whether 2′-5′-(PO or even PS) linkages may provide advantages over the 3′-5′-(PS) linkages currently used in the clinic for terminal stabilization.

In summary, our results demonstrate that 2′-5′/3′-5-siRNAs, when properly designed, can afford a potent class of oligonucleotide therapeutics with diminished immune-stimulatory response. Simplicity, monomer affordability, and feasible synthesis make 2′-5′-linkages an attractive modification to employ in siRNA drug candidates. Based on the structural and functional data provided in this study, it is reasonable to assume that the 2′-5′-linkage modification could be combined with other common modifications (2′OMe, 2′F, PS) or yet to be tested modifications (e.g. 2′-5′-PS) to endow siRNAs with the properties necessary for therapeutic applications. Together with previous studies, our findings on 2′-5′ linkage modification increase our understanding of RNA structure, provide insights for studying their biochemical and prebiotic significance, and help us develop molecules for biomedical applications.

## Supplementary Material

gkaa222_Supplemental_File

## References

[1] Joyce G.F. RNA evolution and the origins of life. Nature. 1989; 338:217–224. 2466202 10.1038/338217a0

[2] Joyce G.F. The antiquity of RNA-based evolution. Nature. 2002; 418:214–221. 12110897 10.1038/418214a

[3] Gilbert W. Origin of life: the RNA world. Nature. 1986; 319:618–618.

[4] Usher D.A., McHale A.H. Nonenzymic joining of oligoadenylates on a polyuridylic acid template. Science. 1976; 192:53–54. 1257755 10.1126/science.1257755

[5] Engelhart A.E., Powner M.W., Szostak J.W. Functional RNAs exhibit tolerance for non-heritable 2′–5′ versus 3′–5′ backbone heterogeneity. Nat. Chem. 2013; 5:390–394. 23609089 10.1038/nchem.1623 PMC4088963

[6] Giannaris P.A., Damha M.J. Oligoribonucleotides containing 2′,5′-phosphodiester linkages exhibit binding selectivity for 3′,5′-RNA over 3′,5′-ssDNA. Nucleic Acids Res. 1993; 21:4742–4749. 7694233 10.1093/nar/21.20.4742 PMC331500

[7] Wasner M., Arion D., Borkow G., Noronha A., Uddin A.H., Parniak M.A., Damha M.J. Physicochemical and biochemical properties of 2′,5′-Linked RNA and 2′,5′-RNA:3′,5′-RNA “Hybrid” duplexes. Biochemistry. 1998; 37:7478–7486. 9585561 10.1021/bi980160b

[8] Sheng J., Li L., Engelhart A.E., Gan J., Wang J., Szostak J.W. Structural insights into the effects of 2′-5′ linkages on the RNA duplex. Proc. Natl. Acad. Sci. U.S.A. 2014; 111:3050–3055. 24516151 10.1073/pnas.1317799111 PMC3939906

[9] Shen F., Luo Z., Liu H., Wang R., Zhang S., Gan J., Sheng J. Structural insights into RNA duplexes with multiple 2′-5′-linkages. Nucleic Acids Res. 2017; 45:3537–3546. 28034958 10.1093/nar/gkw1307 PMC5389462

[10] Prakash T.P., Kraynack B., Baker B.F., Swayze E.E., Bhat B. RNA interference by 2′,5′-linked nucleic acid duplexes in mammalian cells. Bioorg. Med. Chem. Lett. 2006; 16:3238–3240. 16616491 10.1016/j.bmcl.2006.03.053

[11] Damha M.J., Ogilvie K.K. Oligoribonucleotide synthesis. The silyl-phosphoramidite method. Methods Mol. Biol. 1993; 20:81–114. 8242149 10.1385/0-89603-281-7:81

[12] Bellon L. Oligoribonucleotides with 2′-O-(tert-butyldimethylsilyl) groups. Curr. Protoc. Nucleic Acid Chem. 2001; doi:10.1002/0471142700.nc0306s01. 10.1002/0471142700.nc0306s01 18428846

[13] Wincott F., DiRenzo A., Shaffer C., Grimm S., Tracz D., Workman C., Sweedler D., Gonzalez C., Scaringe S., Usman N. Synthesis, deprotection, analysis and purification of RNA and ribozymes. Nucleic Acids Res. 1995; 23:2677–2684. 7544462 10.1093/nar/23.14.2677 PMC307092

[14] Gewirtz A.M., Kalota A., Robert F., Deleavey G.F., Pelletier J., Watts J.K., Sonenberg N., Alain T., Aishwarya V., Damha M.J. Synergistic effects between analogs of DNA and RNA improve the potency of siRNA-mediated gene silencing. Nucleic Acids Res. 2010; 38:4547–4557. 20413581 10.1093/nar/gkq181 PMC2910058

[15] Jahns H., Roos M., Imig J., Baumann F., Wang Y., Gilmour R., Hall J. Stereochemical bias introduced during RNA synthesis modulates the activity of phosphorothioate siRNAs. Nat. Commun. 2015; 6:6317. 25744034 10.1038/ncomms7317 PMC4366519

[16] Gagnon K.T. Loading of argonaute protein with small duplex RNA in cellular extracts. Methods Mol. Biol. 2016; 1421:53–67. 26965257 10.1007/978-1-4939-3591-8_6

[17] Gagnon K.T., Li L., Janowski B.A., Corey D.R. Analysis of nuclear RNA interference in human cells by subcellular fractionation and Argonaute loading. Nat. Protoc. 2014; 9:2045–2060. 25079428 10.1038/nprot.2014.135 PMC4251768

[18] Harikrishna S., Pradeepkumar P.I. Probing the binding interactions between chemically modified siRNAs and human argonaute 2 using microsecond molecular dynamics simulations. J. Chem. Inf. Model. 2017; 57:883–896. 28287733 10.1021/acs.jcim.6b00773

[19] Case D.A., Darden T.A., Cheatham T.E. III, Simmerling C.L., Wang J., Duke R.E.L.R., Walker R.C., Zhang W., Merz K.M., Roberts B. et al. 2014; San Francisco AMBER 14 University of California.

[20] Allnér O., Nilsson L., Villa A. Magnesium Ion–Water coordination and exchange in biomolecular simulations. J. Chem. Theory Comput. 2012; 8:1493–1502. 26596759 10.1021/ct3000734

[21] Meagher K.L., Redman L.T., Carlson H.A. Development of polyphosphate parameters for use with the AMBER force field. J. Comput. Chem. 2003; 24:1016–1025. 12759902 10.1002/jcc.10262

[22] Frisch M.J., Trucks G.W., Schlegel H.B., Scuseria G.E., Robb M.A., Cheeseman J.R., Scalmani G., Barone V., Mennucci B., Petersson G.A. et al. Revision D.01 ed. 2013; Wallingford, CT Gaussian, Inc.

[23] Aduri R., Psciuk B.T., Saro P., Taniga H., Schlegel H.B., SantaLucia J. AMBER force field parameters for the naturally occurring modified nucleosides in RNA. J. Chem. Theory Comput. 2007; 3:1464–1475. 26633217 10.1021/ct600329w

[24] Perez A., Marchan I., Svozil D., Sponer J., Cheatham T.E. 3rd, Laughton C.A., Orozco M. Refinement of the AMBER force field for nucleic acids: improving the description of alpha/gamma conformers. Biophys. J. 2007; 92:3817–3829. 17351000 10.1529/biophysj.106.097782 PMC1868997

[25] Zgarbová M., Otyepka M., Šponer J., Mládek A., Banáš P., Cheatham T.E., Jurečka P. Refinement of the Cornell et al. Nucleic Acids force field based on reference quantum chemical calculations of glycosidic torsion profiles. J. Chem. Theory Comput. 2011; 7:2886–2902. 21921995 10.1021/ct200162x PMC3171997

[26] Cornell W.D., Cieplak P., Bayly C.I., Gould I.R., Merz K.M., Ferguson D.M., Spellmeyer D.C., Fox T., Caldwell J.W., Kollman P.A. A second generation force field for the simulation of proteins, Nucleic Acids, and organic molecules. J. Am. Chem. Soc. 1995; 117:5179–5197.

[27] Junmei W., Piotr C., A. K.P. How well does a restrained electrostatic potential (RESP) model perform in calculating conformational energies of organic and biological molecules. J. Comput. Chem. 2000; 21:1049–1074.

[28] Krepl M., Havrila M., Stadlbauer P., Banas P., Otyepka M., Pasulka J., Stefl R., Sponer J. Can we execute stable Microsecond-Scale atomistic simulations of Protein–RNA complexes. J. Chem. Theory Comput. 2015; 11:1220–1243. 26579770 10.1021/ct5008108

[29] Krepl M., Réblová K., Koča J., Šponer J. Bioinformatics and molecular dynamics simulation study of L1 stalk Non-Canonical rRNA Elements: Kink-Turns, loops, and tetraloops. J. Phys. Chem. B. 2013; 117:5540–5555. 23534440 10.1021/jp401482m

[30] Darden T., York D., Pedersen L. Particle mesh Ewald: An N⋅log(N) method for Ewald sums in large systems. J. Chem. Phys. 1993; 98:10089–10092.

[31] Salomon-Ferrer R., Götz A.W., Poole D., Le Grand S., Walker R.C. Routine microsecond molecular dynamics simulations with AMBER on GPUs. 2. Explicit solvent particle mesh ewald. J. Chem. Theory Comput. 2013; 9:3878–3888. 26592383 10.1021/ct400314y

[32] Le Grand S., Götz A.W., Walker R.C. SPFP: Speed without compromise—A mixed precision model for GPU accelerated molecular dynamics simulations. Comput. Phys. Commun. 2013; 184:374–380.

[33] Berendsen H.J.C., Postma J.P.M., Gunsteren W.F.V., DiNola A., Haak J.R. Molecular dynamics with coupling to an external bath. J. Chem. Phys. 1984; 81:3684–3690.

[34] Roe D.R., Cheatham T.E. PTRAJ and CPPTRAJ: Software for processing and analysis of molecular dynamics trajectory data. J. Chem. Theory Comput. 2013; 9:3084–3095. 26583988 10.1021/ct400341p

[35] Anzahaee M.Y., Watts J.K., Alla N.R., Nicholson A.W., Damha M.J. Energetically important C-H…F-C pseudohydrogen bonding in water: evidence and application to rational design of oligonucleotides with high binding affinity. J. Am. Chem. Soc. 2011; 133:728–731. 21171597 10.1021/ja109817p

[36] Pettersen E.F., Goddard T.D., Huang C.C., Couch G.S., Greenblatt D.M., Meng E.C., Ferrin T.E. UCSF Chimera–a visualization system for exploratory research and analysis. J. Comput. Chem. 2004; 25:1605–1612. 15264254 10.1002/jcc.20084

[37] Hou T., Wang J., Li Y., Wang W. Assessing the performance of the MM/PBSA and MM/GBSA methods. 1. The accuracy of binding free energy calculations based on molecular dynamics simulations. J. Chem. Inf. Model. 2011; 51:69–82. 21117705 10.1021/ci100275a PMC3029230

[38] Deleavey G.F., Damha M.J. Designing chemically modified oligonucleotides for targeted gene silencing. Chem. Biol. 2012; 19:937–954. 22921062 10.1016/j.chembiol.2012.07.011

[39] Frank F., Sonenberg N., Nagar B. Structural basis for 5′-nucleotide base-specific recognition of guide RNA by human AGO2. Nature. 2010; 465:818–822. 20505670 10.1038/nature09039

[40] Khvorova A., Reynolds A., Jayasena S.D. Functional siRNAs and miRNAs exhibit strand bias. Cell. 2003; 115:209–216. 14567918 10.1016/s0092-8674(03)00801-8

[41] Schwarz D.S., Hutvágner G., Du T., Xu Z., Aronin N., Zamore P.D. Asymmetry in the assembly of the RNAi enzyme complex. Cell. 2003; 115:199–208. 14567917 10.1016/s0092-8674(03)00759-1

[42] Conway J.W., McLaughlin C.K., Castor K.J., Sleiman H. DNA nanostructure serum stability: greater than the sum of its parts. Chem. Commun. 2013; 49:1172–1174. 10.1039/c2cc37556g 23287884

[43] Hahn J., Wickham S.F.J., Shih W.M., Perrault S.D. Addressing the instability of DNA nanostructures in tissue culture. ACS Nano. 2014; 8:8765–8775. 25136758 10.1021/nn503513p PMC4174095

[44] Usher D.A., McHale A.H. Hydrolytic stability of helical RNA: a selective advantage for the natural 3′,5′-bond. Proc. Natl Acad. Sci. U.S.A. 1976; 73:1149–1153. 1063396 10.1073/pnas.73.4.1149 PMC430218

[45] Judge A.D., Sood V., Shaw J.R., Fang D., McClintock K., MacLachlan I. Sequence-dependent stimulation of the mammalian innate immune response by synthetic siRNA. Nat Biotech. 2005; 23:457–462. 10.1038/nbt1081 15778705

[46] Hornung V., Guenthner-Biller M., Bourquin C., Ablasser A., Schlee M., Uematsu S., Noronha A., Manoharan M., Akira S., de Fougerolles A. et al. Sequence-specific potent induction of IFN-[alpha] by short interfering RNA in plasmacytoid dendritic cells through TLR7. Nat. Med. 2005; 11:263–270. 15723075 10.1038/nm1191

[47] Egli M., Manoharan M. Re-Engineering RNA molecules into therapeutic agents. Acc. Chem. Res. 2019; 52:1036–1047. 30912917 10.1021/acs.accounts.8b00650

[48] Muhonen P., Tennila T., Azhayeva E., Parthasarathy R.N., Janckila A.J., Vaananen H.K., Azhayev A., Laitala-Leinonen T. RNA interference tolerates 2′-fluoro modifications at the Argonaute2 cleavage site. Chem. Biodivers. 2007; 4:858–873. 17511001 10.1002/cbdv.200790073

[49] Shen X., Corey D.R. Chemistry, mechanism and clinical status of antisense oligonucleotides and duplex RNAs. Nucleic Acids Res. 2018; 46:1584–1600. 29240946 10.1093/nar/gkx1239 PMC5829639

[50] Malek-Adamian E., Guenther D.C., Matsuda S., Martinez-Montero S., Zlatev I., Harp J., Burai Patrascu M., Foster D.J., Fakhoury J., Perkins L. et al. 4′-C-Methoxy-2′-deoxy-2′-fluoro modified ribonucleotides improve metabolic stability and elicit efficient RNAi-Mediated gene silencing. J. Am. Chem. Soc. 2017; 139:14542–14555. 28937776 10.1021/jacs.7b07582

[51] Prakash T.P., Allerson C.R., Dande P., Vickers T.A., Sioufi N., Jarres R., Baker B.F., Swayze E.E., Griffey R.H., Bhat B. Positional effect of chemical modifications on short interference RNA activity in mammalian cells. J. Med. Chem. 2005; 48:4247–4253. 15974578 10.1021/jm050044o

[52] Shukla S., Sumaria C.S., Pradeepkumar P.I. Exploring chemical modifications for siRNA Therapeutics: A structural and functional outlook. ChemMedChem. 2010; 5:328–349. 20043313 10.1002/cmdc.200900444

[53] Bramsen J.B., Pakula M.M., Hansen T.B., Bus C., Langkjaer N., Odadzic D., Smicius R., Wengel S.L., Chattopadhyaya J., Engels J.W. et al. A screen of chemical modifications identifies position-specific modification by UNA to most potently reduce siRNA off-target effects. Nucleic Acids Res. 2010; 38:5761–5773. 20453030 10.1093/nar/gkq341 PMC2943616

[54] Schirle N.T., Sheu-Gruttadauria J., MacRae I.J. Structural basis for microRNA targeting. Science (New York, N.Y.). 2014; 346:608–613. 10.1126/science.1258040 PMC4313529 25359968

[55] Schirle N.T., MacRae I.J. The crystal structure of human Argonaute2. Science (New York, N.Y.). 2012; 336:1037–1040. 10.1126/science.1221551 PMC3521581 22539551

[56] Lu X.J., Olson W.K. 3DNA: a software package for the analysis, rebuilding and visualization of three‐dimensional nucleic acid structures. Nucleic Acids Res. 2003; 31:5108–5121. 12930962 10.1093/nar/gkg680 PMC212791

[57] Lu X.-J., Olson W.K. 3DNA: a versatile, integrated software system for the analysis, rebuilding, and visualization of three-dimensional nucleic-acid structures. Nat. Protoc. 2008; 3:1213–1227. 18600227 10.1038/nprot.2008.104 PMC3065354

[58] Gagnon K.T., Li L., Chu Y., Janowski B.A., Corey D.R. RNAi factors are present and active in human cell nuclei. Cell Rep. 2014; 6:211–221. 24388755 10.1016/j.celrep.2013.12.013 PMC3916906

[59] Nichols J.G., Crooke S.T., Lima W.F., Vickers T.A. Reduced levels of Ago2 expression result in increased siRNA competition in mammalian cells. Nucleic Acids Res. 2007; 35:6598–6610. 17905815 10.1093/nar/gkm663 PMC2095815

[60] Agrawal S., Gait M.J. Advances in Nucleic Acid Therapeutics. 2019; The Royal Society of Chemistry 1–21.

[61] Shen X., Corey D.R. Chemistry, mechanism and clinical status of antisense oligonucleotides and duplex RNAs. Nucleic Acids Res. 2017; 46:1584–1600. 10.1093/nar/gkx1239 PMC5829639 29240946

[62] Crooke S.T. Molecular mechanisms of antisense oligonucleotides. Nucleic Acid Ther. 2017; 27:70–77. 28080221 10.1089/nat.2016.0656 PMC5372764

