# Supplementary material for: Effect of 2′-5′/3′-5′ phosphodiester linkage heterogeneity on RNA interference
Source: Nucleic Acids Res. 2020 Apr 13;48(9):4643–57. doi: 10.1093/nar/gkaa222 (PMC7229817; doi:10.1093/nar/gkaa222)
Supplement: gkaa222_Supplemental_File [file gkaa222_supplemental_file.pdf]

Supplementary Information

Effect of 2′-5′/3′-5′ phosphodiester linkage heterogeneity on RNA interference

Maryam Habibian[1], S. Harikrishna[2], Johans Fakhoury[1], Maria Barton[3], Eman A. Ageely[4], Regina Cencic[5], Hassan Fakih[1], Adam Katolik[1], Mayumi Takahashi[6], John Rossi[6], Jerry Pelletier[5], Keith T. Gagnon[3,4], P.I. Pradeepkumar[2], and Masad J. Damha[1,*]

[1]Department of Chemistry, McGill University, 801 Sherbrooke St. West, Montreal, QC H3A 0B8, Canada
[2] Department of Chemistry, Indian Institute of Technology Bombay, Mumbai−400076, India
[3] Department of Biochemistry and Molecular Biology, Southern Illinois University School of Medicine, Carbondale, IL, USA
[4] Department of Chemistry and Biochemistry, Southern Illinois University, Carbondale, IL, USA
[5] Department of Biochemistry and Goodman Cancer Center, McGill University, Montreal, QC, H3G 1Y6, Canada
[6] Department of Molecular and Cellular Biology, Beckman Research Institute of City of Hope, Duarte, CA, USA

* To whom correspondence should be addressed. Tel: +1514-398-7552; Fax: +1514-398-3797; Email: masad.damha@mcgill.ca

SUPPLEMENTARY FIGURES

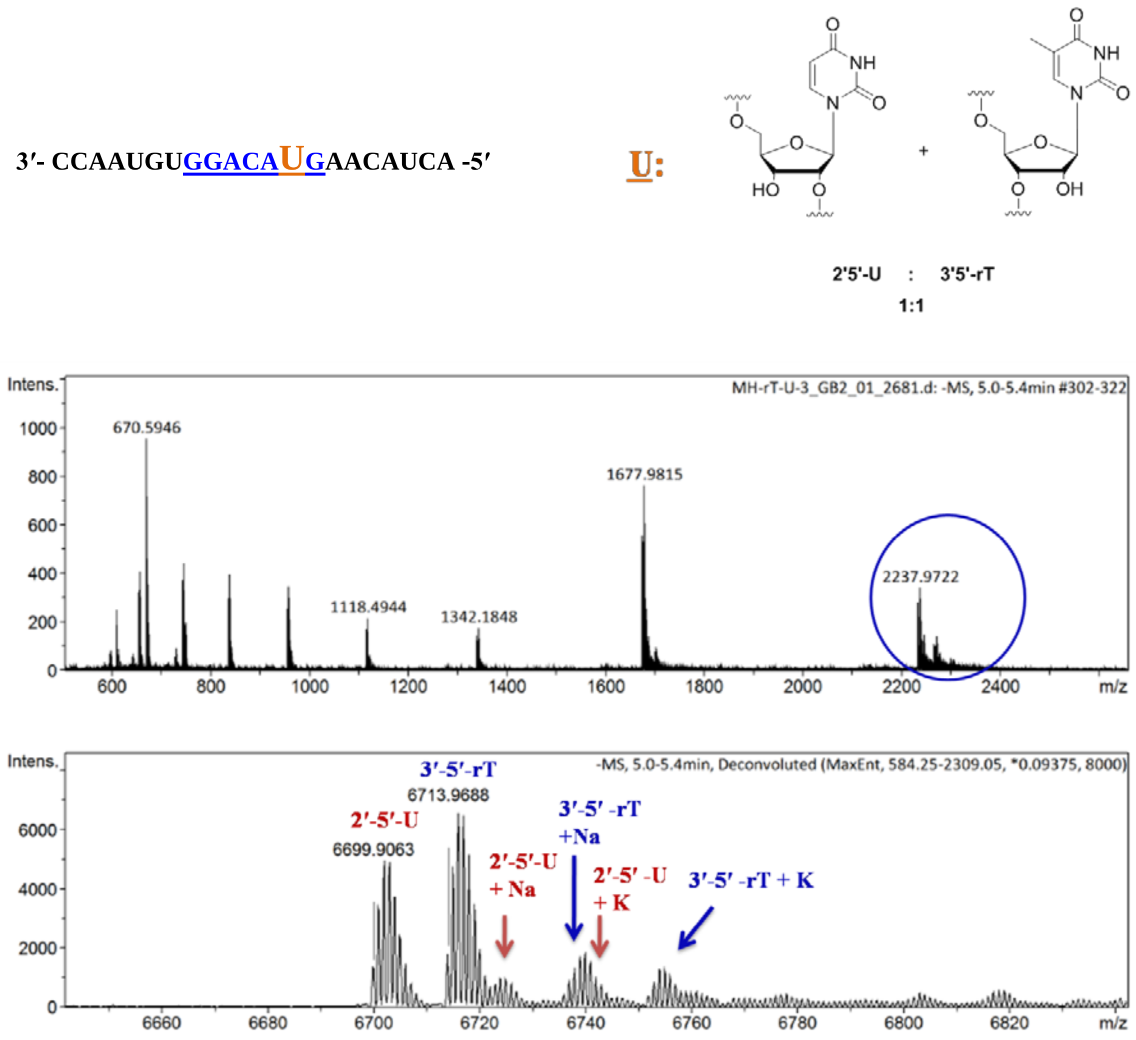

Figure S1. Assessing the binding efficiency of 2'P amidites versus 3'P amidites during a mixmer synthesis using ETT as activator. The sequence (top) shows the position of the mixmer segment (mixmer) and the rU/rT mixmer (U). HRMS profile of a test mixmer is shown. The circled peak envelope represents the (-3) charge state, which is deconvoluted to the peaks shown in the bottom spectrogram. Each peak envelope is assigned with the species it corresponds to within the mixmer product. Assessment of the areas under the peaks show the relative coupling efficiency of 0.77:1 for 2'-5':3'-5' RNA amidites while using ETT as an activator.

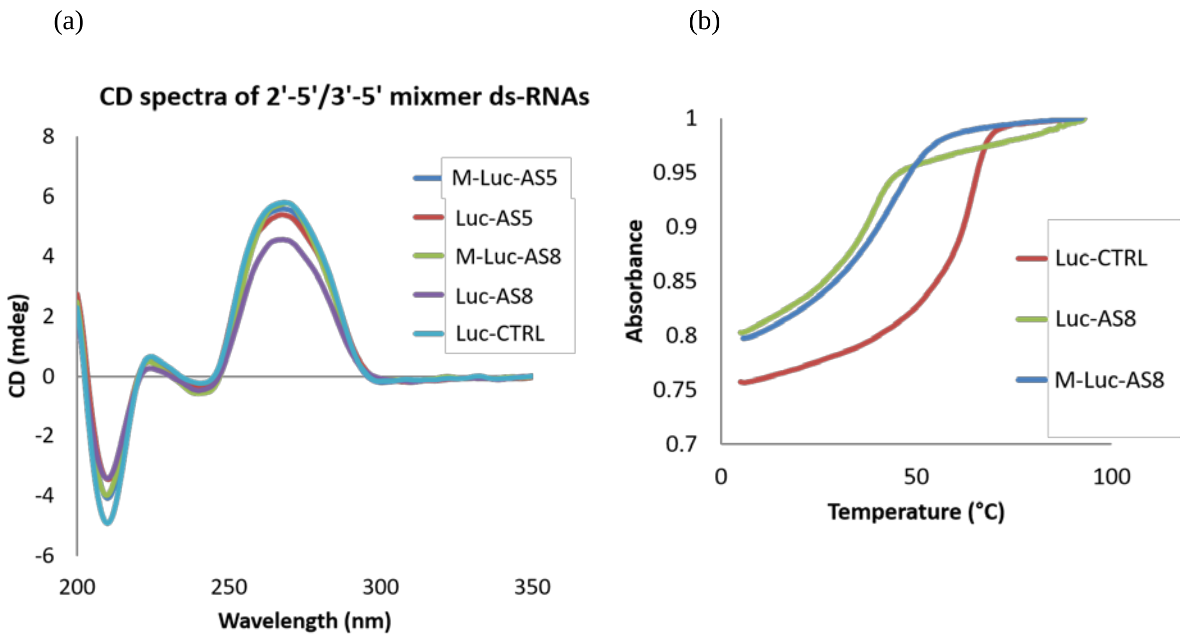

M-Luc-AS5	5'- GCUUGAA GUCUUUA AUUAAUU -3'<br>3'- GGCGAAC UUCAGAA AUUAAUU -5'
Luc-AS5	5'- GCUUGAA GUCUUUA AUUAAUU -3'<br>3'- GGCGAAC UUCAGAA AUUAAUU -5'
M-Luc-AS8	5'- GCUUGAAGUCUUUAAUUAAUU -3'<br>3'- GGCGAAC UUCAGAA AUUAAUU -5'
Luc-AS8	5'- GCUUGAA GUCUUUA AUUAAUU -3'<br>3'- GGCGAACUUCAGAAAUUAAUU -5'
Luc- ctrl	5'- GCUUGAA GUCUUUA AUUAAUU -3<br>3'- GGCGAAC UUCAGAA AUUAAUU -5'

Figure S2. Normalized CD spectra and UV melting profiles of selected 2'-5'/3'-5' mixmer and 2'-5' modified siRNAs targeting firefly luciferase. Sample sequences and modification patterns are presented above. Melting profiles of selected siRNAs targeting firefly luciferase with 2'-5' or 2'-5'/3'-5' mixmer modification placed in the sense or antisense strands were followed by observing the change in absorbance at 260 nm (A260) of duplex samples upon heating from 5°C to 90°C. Duplexes were 1.5μM (3μM total concentration of strands) in phosphate buffer (140mM KCl, 5mM Na_2HPO4, 1mM $MgCl_2$, pH 7.2) Legend: 2'-5'/3'-5' mixmer linked RNA, 2'-5' linked RNA, 3'-5' linked RNA.

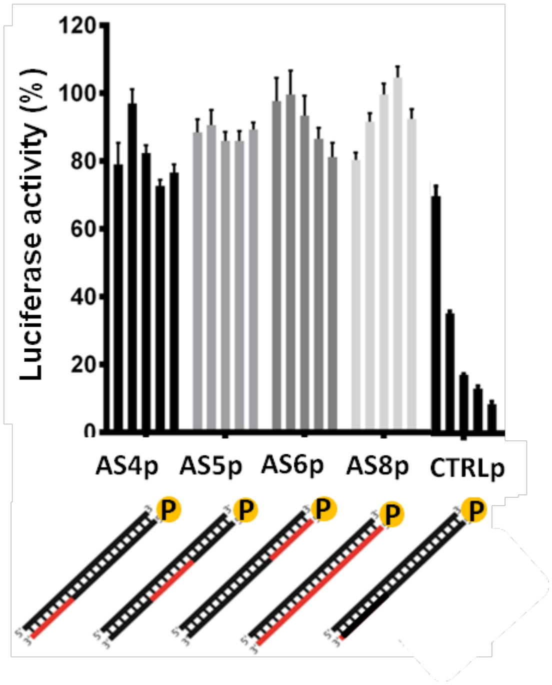

Figure S3. Assay demonstrating the activity of siRNAs AS4p, AS5p, AS6p, AS8p, and unmodified CTRL (control) with 2'-5' modification on their AS strand targeting firefly luciferase mRNA. All the antisense strands are 5'-phosphorylated chemically to bypass enzymatic phosphorylation by kinase. The red segments in the duplex illustrations represent the position of the 2'-5' modification within the antisense strand. "P" represents the 5'-PO group. siRNA sequences are shown in Figure 2a.

FIGURE S4. Calculation of the band intensities and % of duplexes E, F and G remaining. Intensities of bands (50% FBS) shown in a) were quantified using the Image Lab 5.2 software, and the values for 'ratio 1' and the % duplex remaining (given in (b) below) were calculated using the equation provided in the Materials and Method Section. c) PAGE of single stranded and duplexed RNAs in the absence of FBS. The same analyses were applied at duplexes treated with 10% FBS (d), and SPDE (e). Codes: 3′-5′-phosphorothioate linkages (blue rectangles; duplex G), 2′-5′ linkages (red rectangles; duplexes E and F), all other residues are linked via 3′-5′ linkages.

a)

ID	Duplex Design	T_m (°C)
E	5′ GGUGGAAUCUCUGGUAUCUTT 3′<br>3′ TUCCACCUUAGAGACCAUAGA 5′	73.5
F	5′ GGUGGAAUCUCUGGUAUCUTT 3′<br>3′ TUCCACCUUAGAGACCAUAGA 5′	67.0
G	5′ GGUGGAAUCUCUGGUAUCUTT 3′<br>3′ TUCCACCUUAGAGACCAUAGA 5′	74.8

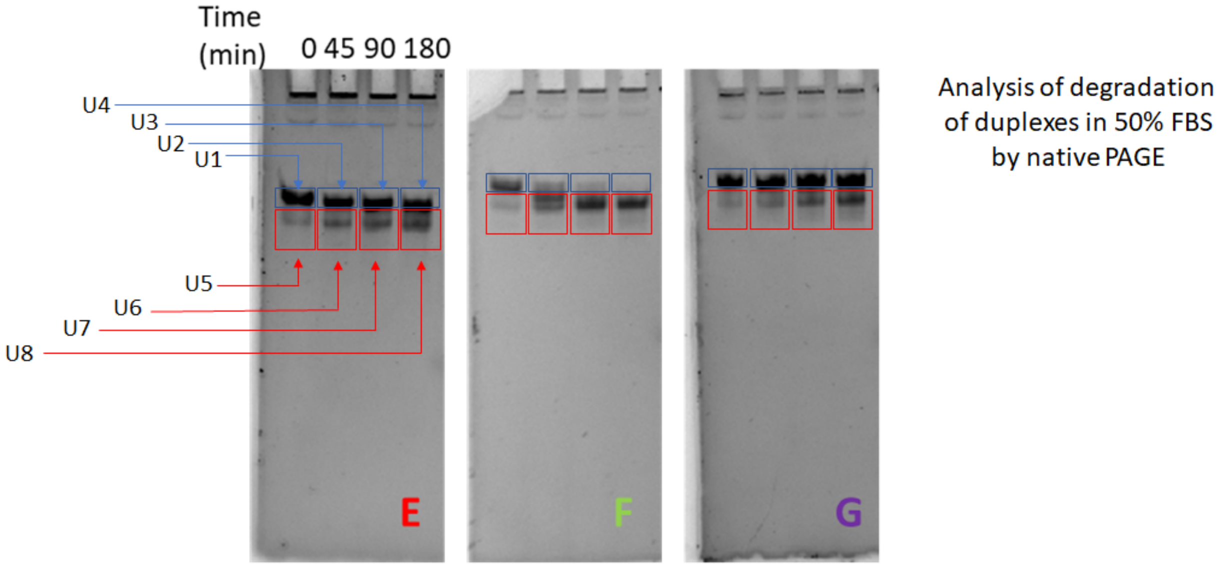

FIGURE S4 –cont'd

b)

FBS assay data

Duplex E				
**Time (min)**	**SM (a.u)**	**DP (a.u)**	**Ratio 1**	**% D. R.**
0	10,644,161.00	6,293,729.00	1.691233	100
45	8,814,165.00	7,279,394.00	1.210838	71.59498
90	8,736,028.00	7,931,274.00	1.101466	65.12799
180	7,167,577.00	8,420,044.00	0.851252	50.33321

Duplex F				
**Time (min)**	**SM (a.u)**	**DP (a.u)**	**Ratio 1**	**% D. R.**
0	4,176,334.00	3,032,596.00	1.377148	100
45	2,755,662.00	5,707,029.00	0.482854	35.06188
90	2,106,449.00	7,450,659.00	0.28272	20.52937
180	1,867,825.00	7,564,896.00	0.246907	17.92886

Duplex G				
**Time (min)**	**SM (a.u)**	**DP (a.u)**	**Ratio 1**	**% D. R.**
0	7,882,437.00	5,501,237.00	1.432848	100
45	8,914,108.00	6,283,397.00	1.418677	99.01095
90	10,212,216.00	7,310,813.00	1.396865	97.48867
180	9,099,233.00	8,517,123.00	1.068346	74.561

c) PAGE of single stranded and duplexed RNAs in the absence of FBS.

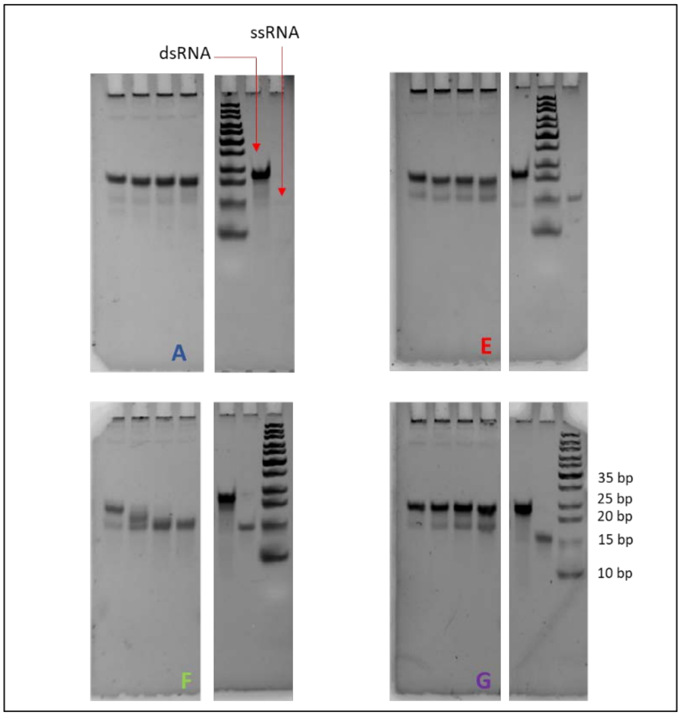

FIGURE S4 –cont'd

d) Digestion of duplexes with 10% FBS

ID	Duplex Design	T_m (ºC)
A	5′ GGUGGAAUCUCUGGUAUCUTT 3′<br>3′ TUCCACCUUAGAGACCAUAGA 5′	75.2
E	5′ GGUGGAAUCUCUGGUAUCUTT 3′<br>3′ TUCCACCUUAGAGACCAUAGA 5′	73.5
F	5′ GGUGGAAUCUCUGGUAUCUTT 3′<br>3′ TUCCACCUUAGAGACCAUAGA 5′	67.0
G	5′ GGUGGAAUCUCUGGUAUCUTT 3′<br>3′ TUCCACCUUAGAGACCAUAGA 5′	74.8

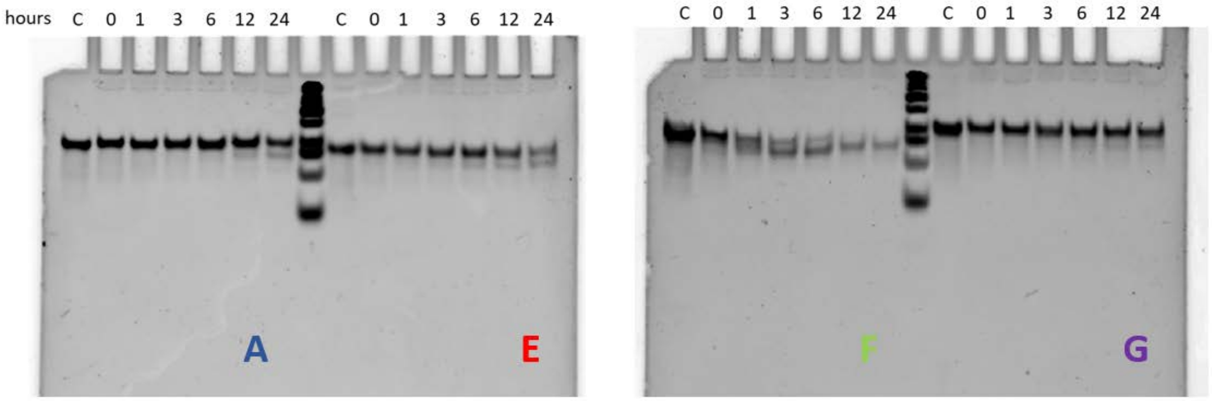

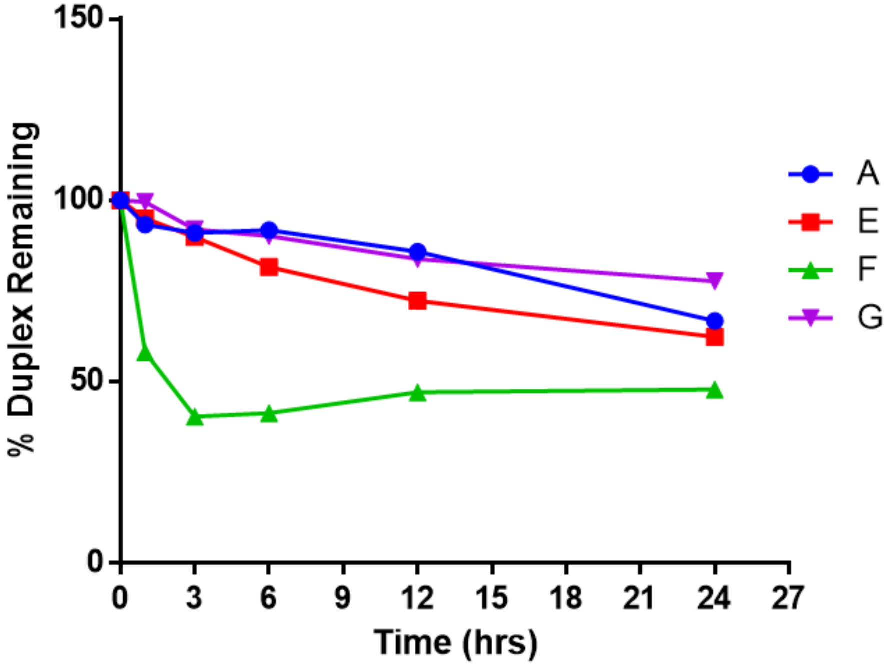

e)

Digestion of duplexes with Spleen Phosphodiesterase (SPDE)

ID	Duplex Design	T_m (°C)
E	5′ G G U G G A A U C U C U G G U A U C U T T 3′<br>3′ T U C C A C C U U A G A G A C C A U A G A 5′	73.5
F	5′ G G U G G A A U C U C U G G U A U C U T T 3′<br>3′ T U C C A C C U U A G A G A C C A U A G A 5′	67.0
G	5′ G G U G G A A U C U C U G G U A U C U T T 3′<br>3′ T U C C A C C U U A G A G A C C A U A G A 5′	74.8

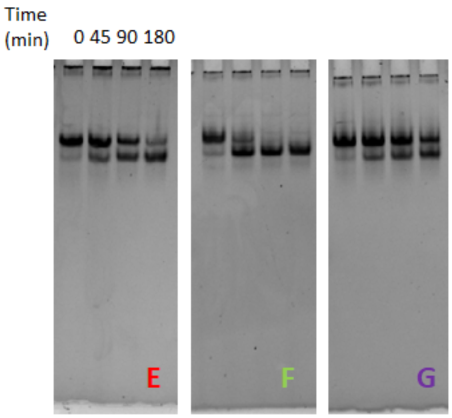

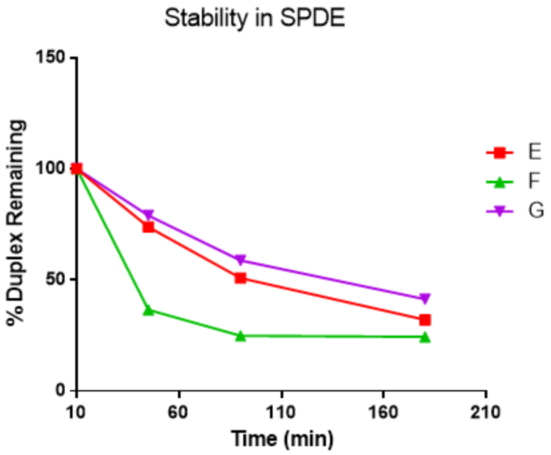

Enzyme source: https://www.sigmaaldrich.com/catalog/product/sigma/p9041?lang=en&region=CA

SPDE (10 units) was dissolved in DEPC-water, and then 0.4 units was added to each duplex (75 pmoles) in 100mM NaOAc (pH = 6.5) and incubated at 37 °C.

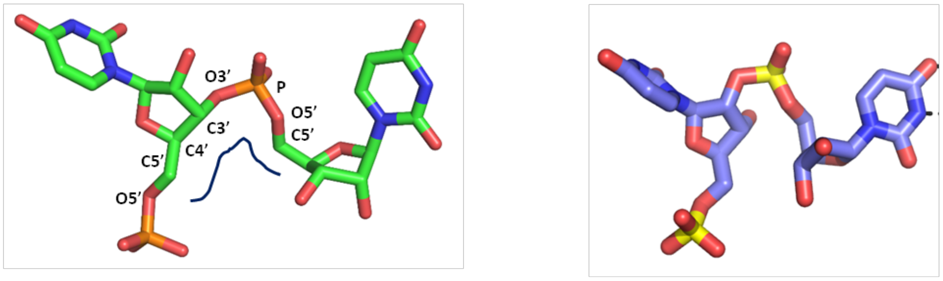

3′-5′ linked **2′-5′ linked**

Figure S5. Clover leaf junction in an unmodified 3'-5' linked (left) versus a 2'-5' linked (right) siRNA. Atoms comprising the dihedral angels are labelled. Clover leaf junction is illustrated by a dark line.

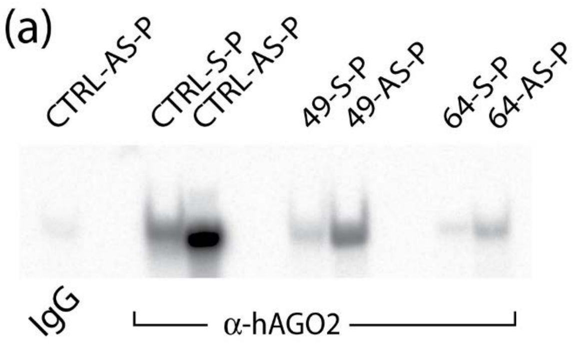

Figure S6. Gel image representing the hAGO2 loading assay using radiolabelled siRNAs. Loading levels of a selected library of 2',5' modified siRNAs (#49 and #64) as well as the unmodified control siRNA in hAGO2 was analysed in gel shift assay. Details of represented siRNAs and graphs are provided in Figure 9.

SUPPLEMENTARY TABLES

Table S1. MS characterization of S and AS strands comprising the siRNAs targeting firefly luciferase, P53, and 728UU (All analyzed in negative ion mode). 2′-5′ linkage modified samples as well as all the mixmers have the same mass as their unmodified 3′,5′ RNA counterparts.

Sample Name	Sequence	Exact Mass
Luciferase S	5'- GCUUGAAGUCUUUAAUUAAUU -3'	6617.8352
Luciferase AS	3'- GGCGAACUUCAGAAAUUAAUU-5'	6701.9292
Luciferase AS-P	3'- GGCGAACUUCAGAAAUUAAUU-5' (PO_4)	6780.8877
P53 S	3'-CCAAUGUGUACAUGAACAUCA-5'	6660.9392
P53 AS	5'-UUACACAUGUACUUGUAGUGG-3'	6671.8672
728UU S	5'- AAAUCGCUGAUUUGUGUAGUU-3'	6672.8512
728UU AS	3'-UUUUUAGCGACUAAACACAUC-5'	6598.8892

Table S2. Dihedral angle adopted by the unmodified and modified siRNAs at the junction of g1 and g2 (clover leaf model).[a]

Unmodified	Dihedral angle values	Modified	Dihedral angle values (49)	Dihedral angle values (54)
O5'-C5'-C4'-C3'	-173.5	O5'-C5'-C4'-C3'	-179.8	-172.3
C5'-C4'-C3'-O3'	-143.8	C4'-C3'-C2'-O2'	-150.7	-155.5
C4'-C3'-O3'-P	-146.7	C3'-C2'-O2'-P	-45.6	-43.9
C3'-O3'-P-O5'	53.6	C2'-O2'-P-O5'	-38.3	-41.2
O3'-P-C5'-O5'	65.8	O2'-P-C5'-O5'	120.5	127.6
P-O5'-C5'-C4'	154.7	P-O5'-C5'-C4'	-160.6	-156.4
C5'-C4'-C3'-O3'	41.6	C5'-C4'-C3'-O3'	-33.9	-41.7

[a]All the values are mentioned in degree and computed using X3DNA package.

Table S3. Percentage occupancy of W-C H-bond in the unmodified and modified siRNAs.[a]

Base pairs	Unmodified	Modified siRNA (49)	Modified siRNA (64)
U-A	99.8%	46.8%	43.5%
A-U	99.9%	97.8%	86.9%
A-U	99.6%	98.7%	39.7%
U-A	99.8%	99.8%	76.3%
U-A	98.9%	99.6%	35.4%
A-U	99.3%	99.3%	78.3%

[a] Calculations were carried out using CPPTRAJ module in AMBER 14 package.

Table S4. Binding free energy components for siRNA-hAGO2 complexes calculated from the last 500 ns of the 1 μs MD simulations. [a]

Calculated value	Unmodified	siRNA49	siRNA64
ΔE_{ELEC}	−2444 ± 25	−2356± 21	−2210 ± 23
ΔE_{VDW}	−313 ± 9	−285 ± 11	−208 ± 9
ΔE_{MM}	−2757± 23	−2641± 26	−2418± 29
ΔG_{PB}	2463± 25	2389± 25	2492± 22
ΔG_{NP}	−13± 0.5	−13 ± 0.5	−13 ± 0.6
ΔG_{SOLV}	2450 ± 21	2376 ± 21	2589 ± 21
ΔH_{PB}	−307± 8	−265± 9	−171± 9
$T\Delta S$	−58 ± 3	−54 ± 3	−46 ± 3
ΔG ($\Delta\Delta G$)	−249 ± 7	−211 ± 11 (38 ± 9)	−125 ± 12 (124 ± 9)

[a]All the values are mentioned in kcal/mol. ΔE_{ELEC}, electrostatic energy; ΔE_{VDW}, van der Waals energy; ΔE_{INT} internal energy is negligible in all the cases; $\Delta E_{MM} = \Delta E_{ELEC} + \Delta E_{VDW} + \Delta E_{INT}$; ΔG_{PB}, polar solvation free energy; ΔG_{NP}, non-polar solvation free energy; $\Delta G_{SOLV} = \Delta G_{PB} + \Delta G_{NP}$; $\Delta H_{PB} = \Delta E_{MM} + \Delta G_{SOLV}$; $T\Delta S$, total entropy contribution; $\Delta G = \Delta H_{PB} - T\Delta S$, total binding free energy; $\Delta\Delta G = \Delta G_{modified} - \Delta G_{unmodified}$, difference in the binding free energy. Averaged over 1000 snapshots. All these values are determined using MM-PBSA approach,[49] entropy contributions using normal mode analysis.
